# Metabolic characteristics and pathogenesis of precocious puberty in girls: the role of perfluorinated compounds

**DOI:** 10.1186/s12916-023-03032-0

**Published:** 2023-08-25

**Authors:** Jinxia Wu, Jing Chen, Rong Huang, Hongwei Zhu, Lin Che, Yanyan Lin, Yajie Chang, Guiping Shen, Jianghua Feng

**Affiliations:** 1 Department of Electronic Science, Fujian Provincial Key Laboratory of Plasma and Magnetic Resonance, Xiamen University, Siming District, 422 Siming South Road, Xiamen, 361005 Fujian China; 2 Department of Child Health, Women and Children’s Hospital, School of Medicine, Xiamen University, Xiamen, 361003 Fujian China; 3 Department of Pediatrics, The First Affiliated Hospital of Bengbu Medical College, Anhui, Bengbu, 233000 China; 4 Sun Yat-Sen University Cancer Center, State Key Laboratory of Oncology in South China, Guangzhou, 510060 Guangdong China

**Keywords:** Premature thelarche, Central precocious puberty, Specific biomarkers, Metabolic network, Perfluorinated compounds

## Abstract

**Background:**

Precocious puberty (PP) in girls is traditionally defined as the onset of breast development before the age of 8 years. The specific biomarkers of premature thelarche (PT) and central precocious puberty (CPP) girls are uncertain, and little is known about their metabolic characteristics driven by perfluorinated compounds (PFCs) and clinical phenotype. This study aimed to screen specific biomarkers of PT and CPP and elucidate their underlying pathogenesis. The relationships of clinical phenotype-serum PFCs-metabolic characteristics were also explored to reveal the relationship between PFCs and the occurrence and development of PT and CPP.

**Methods:**

Nuclear magnetic resonance (NMR)-based cross-metabolomics strategy was performed on serum from 146 PP (including 30 CPP, 40 PT, and 76 unspecified PP) girls and 64 healthy girls (including 36 prepubertal and 28 adolescent). Specific biomarkers were screened by the uni- and multivariate statistical analyses. The relationships between serum PFCs and clinical phenotype were performed by correlation analysis and weighted gene co-expression network analysis to explore the link of clinical phenotype-PFCs-metabolic characteristics in PT and CPP.

**Results:**

The disordered trend of pyruvate and butyrate metabolisms (metabolites mapped as formate, ethanol, and 3-hydroxybutyrate) were shared and kept almost consistent in PT and CPP. Eight and eleven specific biomarkers were screened for PT and CPP, respectively. The area under curve of specific biomarker combination was 0.721 in CPP *vs*. prepubertal, 0.972 in PT *vs*. prepubertal, 0.646 in CPP *vs*. prepubertal integrated adolescent, and 0.822 in PT *vs*. prepubertal integrated adolescent, respectively. Perfluoro-n-heptanoic acid and perfluoro-n-hexanoic acid were statistically different between PT and CPP. Estradiol and prolactin were significantly correlated with PFCs in CPP and PT. Clinical phenotypes and PFCs drive the metabolic characteristics and cause metabolic disturbances in CPP and PT.

**Conclusions:**

The elevation of formate, ethanol, and 3-hydroxybutyrate may serve as the early diagnostic indicator for PP in girls. But the stratification of PP still needs to be further determined based on the specific biomarkers. Specific biomarkers of CPP and PT exhibited good sensitivity and can facilitate the classification diagnosis of CPP and PT. PFC exposure is associated with endocrine homeostasis imbalance. PFC exposure and/or endocrine disturbance directly or indirectly drive metabolic changes and form overall metabolic network perturbations in CPP and PT.

**Supplementary Information:**

The online version contains supplementary material available at 10.1186/s12916-023-03032-0.

## Background

Pubertal timing is usually regulated by complex interplay of genetic, environmental, nutritional, and epigenetic factors. Therefore, the criteria for normal pubertal timing and thus the definition of precocious puberty are hard to determine. Precocious puberty (PP) in girls is traditionally defined as the onset of breast development before the age of 8 years [1]. Its underlying pathophysiology may be gonadotropin-releasing hormone (GnRH)-dependent for central precocious puberty (CPP) girls or GnRH-independent for premature thelarche (PT) girls. CPP is mainly induced by the continuous pulse secretion of GnRH to prematurely activate the hypothalamic-pituitary-gonadal (HPG) axis; however, the exact mechanisms remain unclear. The main clinical manifestation of the PT girls is simple breast development due to exposure to the peripheral estrogen environment. When PT is accompanied by the significant advance growth of bone age, it is more likely to evolve into secondary CPP. CPP can lead to short- and long-term complications in girls, including increased risk of psychosocial distress, short stature, obesity, cardiovascular disease, and type 2 diabetes in adulthood [2]. Therefore, it is vital to understand the etiology of PT and CPP for accurate diagnosis and prompt intervention.

Some researchers tried to quantify PP with the help of clinical phenotype such as luteinizing hormone (LH), follicular-stimulating hormone (FSH), and estradiol to determine the index threshold for CPP diagnosis, but it is still confronted with great controversy and challenge at this moment [3, 4]. Some evidences have indicated the changes of metabolic profile during puberty. Qi et al. found that catecholamine metabolic pathway, tryptophan metabolic pathway, and TCA cycle were disturbed in CPP girls by GC/LC-MS-based urinary metabolomics analysis [5]. Yang et al. used LC-MS technology to characterize the urinary metabolomes of CPP girls and found that amino acids, especially aromatic amino acids, were closely related to the pathogenesis of CPP by activating the HPG axis and inhibiting the hypothalamic-pituitary-adrenal axis [6]. However, the clinical differential diagnosis of CPP and PT is still in a vague interface, and the lack of powerful molecular biomarkers is a long-term bottleneck in the clinical diagnosis and evaluation of PP.

Recently, ubiquitous exposures to polyfluorinated compounds (PFCs) have attracted concerns regarding their possible harmful effects during critical periods of development in early-life and long-term consequences on health in consideration of their persistence and bioaccumulation potential. Massive researches have shown that PFCs can interfere with estrogen homeostasis and pose a risk of endocrine-disrupting effects [7–9], and they are association with dyslipidemia, renal function, and age at menarche [7, 10–12], but there is still inconsistency in the research results [13] as well as certain gender differences [14–17]. In addition, evidences have shown that PFCs can affect the HPG axis [18, 19] or directly affect the gonad axis through their weak estrogen or antiandrogen effects to disrupt the development of puberty [20]. However, the correlation research of PFC exposure with the occurrence and development of PP is still in its infancy [19, 21, 22], and therefore extensive in-depth research and exploration is urgently required to clarify the exact response mechanism. The correlation analysis between PFCs and the clinical phenotype in girls with PP as well as the endogenous metabolites driven by PFCs will help to reveal the impact of PFCs on the occurrence and development of precocious puberty in girls and the preliminary mechanisms.

Based on this, the serum metabolic profiles of prepubertal, PP, PT, CPP, and adolescent girls were characterized by one-dimensional nuclear magnetic resonance hydrogen spectrum (^1^H-NMR) technology, and the metabolic differences and connections were analyzed by cross-metabolomics analyses, aiming to screen the specific biomarkers of CPP and PT. Furthermore, to reveal the effect of PFCs on the occurrence of CPP and PT, the metabolic modules driven by PFCs and clinical phenotype were identified by weighted gene co-expression network analysis (WGCNA).

## Methods

### Subject selection and sampling

**Fig. 1:**
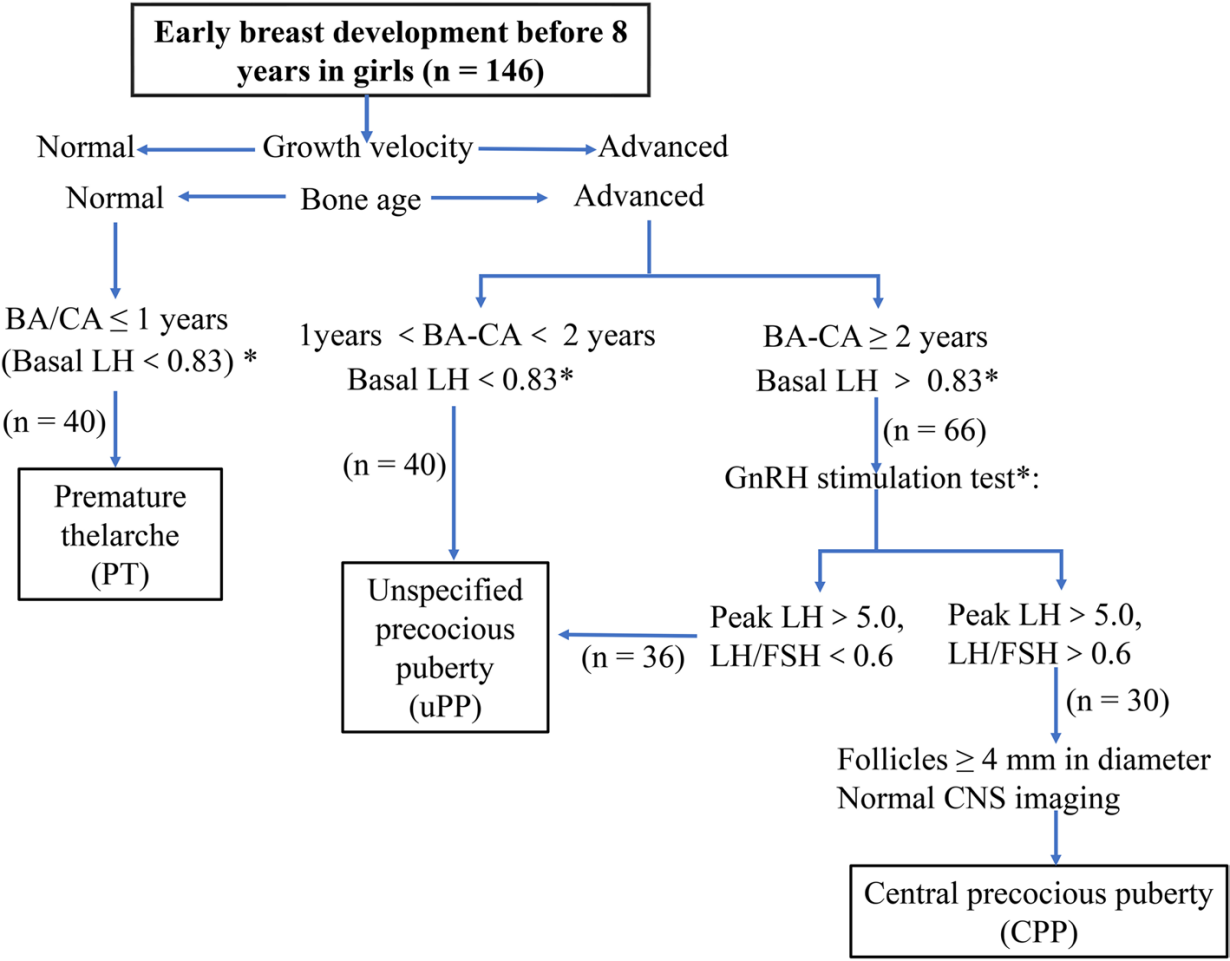
Inclusion and screening process for study cohorts. *: Depending on the method of measurement. The detection method of basal LH and GnRH stimulation test in this study was immunochemiluminometric. LH: luteinizing hormone; FSH: follicular-stimulating hormone; BA: bone age (at the time of diagnosis); CA: chronologic age (at the time of diagnosis)

The children were enrolled from the Department of Child Health, Women and Children’s Hospital, School of Medicine, Xiamen University. A total of 146 PP (including 30 CPP, 40 PT, and 76 unspecified PP) girls were enrolled at their first visit. The inclusion criteria of the patients are shown in detail in Fig. 1 according to the clinical guidelines and the related literatures [23–25]. In addition, 64 healthy girls were recruited as a control group for metabolic comparison, who were divided into 36 prepubertal and 28 adolescent girls based on their developmental status. Relevant clinical phenotypes were collected during the clinical examination. Morning fasting serum sample was collected from each girl through a clinical standard procedure and stood for 30 min, then centrifuged at 1000*g* for 10 min at 4 °C. The serum supernatant was transferred to a new centrifuge tube and stored at −80 °C until analysis.

### Sample preparation, ^1^H-NMR spectra acquisition and processing

All serum samples were thawed at 4 °C, and 400 μL of serum was mixed with 200 μL of 60 mM phosphate buffer (pH 7.4, in 0.9% deuterated saline solution) and then vortexed for 10 s. After being centrifuged at 13,000*g* for 10 min at 4 °C, 550 μL of supernatant was transferred into a 5-mm NMR tube for ^1^H-NMR spectral acquisition.

The ^1^H-NMR spectra of serum samples were obtained on a 600-MHz Bruker Advance nuclear magnetic resonance (NMR) spectrometer (Bruker BioSpin, Germany) equipped with a triple resonance cryogenic probe operating at 600.13 MHz and 298.0 K. A typical water-suppressed Carr-Purcell-Meiboom-Gill (CPMG, [RD-90°-(τ-180°-τ)_n_-ACQ]) pulse sequence with a spectral width of 12,019.2 Hz, an acquisition time of 1.36 s, a relaxation delay of 4.0 s, a scan accumulation of 64 times, and a data point of 16 K was adopted to acquire ^1^H-NMR spectra.

Spectral processing was performed on MestReNova (version 14.1.1, Mestrelab Research S.L., Spain). All the free induction decays were zero-filled to 64 K data points and multiplied by an exponential function of 1.0 Hz line-broadening factor. The ^1^H-NMR spectra were manually phased, and baseline corrected, and then referenced to the doublet of endogenous lactate at δ1.33 after Fourier transformation. The spectral regions of δ4.70–δ5.17 and δ5.50–δ6.00 were removed to eliminate the interference of residual aquatic and urea signals. The remainder spectral regions (δ0.55–δ8.60) were integrally segmented into discrete regions of 0.002 ppm. To reduce the concentration difference between the samples, the obtained NMR spectral data were normalized to the total integrated area.

### LC-MS/MS detection of serum PFCs

The serum PFCs of 40 PT and 30 CPP girls were measured by LC-MS/MS technique, and eleven kinds of PFCs, including perfluoro-n-octanoic acid (PFOA), potassium perfluoro-1-octanesulfonate (PFOS), perfluoro-n-butanoic acid (PFBA), perfluoro-n-undecanoic acid (PFUnDA), perfluoro-n-dodecanoic acid (PFDoDA), potassium perfluoro-1-butanesulfonate (PFBS), perfluoro-n-decanoic acid (PFDA), perfluoro-n-heptanoic acid (PFHpA), perfluoro-n-hexanoic acid (PFHA), potassium perfluoro-1-hexanesulfonate (TFHSA), and perfluoro-n-nonanoic acid (PFNA), were detected according to the references [26, 27]. The detailed procedures of serum sample preparation and PFC detection are shown in section S1 and Figure S1 in the Additional file 1.

### Data processing and statistical analysis

The data are expressed as the means ± standard deviation (S.D.). All univariate statistical analysis was carried out using SPSS 20.0 software (SPSS Inc., Chicago, IL). According to distribution and variance homogeneity of indicator variables of data, Student’s *t* test or Mann-Whitney test were used for comparisons between two groups. If the variance was homogeneous, the *p* value was calculated directly; otherwise, Welch-Satterthwaite method was used. Differences were considered statistically significant when *p* < 0.05. Linear regression analysis was performed to correct the age factors.

The processed data of NMR were used for multivariate statistical analysis on SIMCA 14.1 software (Umetrics, Umea, Sweden) including principal component analysis (PCA) and orthogonal partial least squares-discriminant analysis (OPLS-DA). The normalized dataset was scaled by unit variance, which makes each variable have the same variance. PCA is usually used for variable reduction and display of the relationship between samples such as whether there is clustering or outlier. The OPLS-DA models were applied to maximize the metabolic differences and extract the differential metabolites between the pair-wise groups. The NMR signals were assigned to individual metabolites with the referenced proton NMR peaks from Chenomx NMR Suite 8.1 (Chenomx Inc., EDBonton, AB, Canada) and confirmed by the public Human Metabolome Database (HMDB) (http://www.hmdb.ca/). In this study, the potential biomarkers were screened according to the following criteria: the value of variable importance for projection (VIP) > 1 of the metabolite and the *p* value after age correction (p-adj) < 0.05.

The comprehensive metabolic network was constructed by integrating all potential biomarkers identified from the present research using the Kyoto Encyclopedia of Genes and Genomes (KEGG) (http://www.genome.ad.jp/kegg/), HMDB (http://www.hmdb.ca/) and MetaboAnalyst 5.0 (https://www.metaboanalyst.ca/). Sensitivity and specificity of the potential biomarkers were analyzed by an exploratory receiver operating characteristic (ROC) curve based on random forest, and the area under ROC curve (AUC) and confidence interval (Cl) were determined correspondingly.

For the serum PFCs, after the raw data were transformed by natural logarithm, the Mann-Whitney test was utilized to analyze the statistical difference between PT and CPP girls. Spearman correlation analysis was performed to analyze the association of PFCs and clinical phenotype in PT and CPP girls, respectively.

### WGCNA procedure

In order to better understand the relationships of clinical phenotype-PFCs-metabolic characteristics, WGCNA were further used to identify the metabolite modules driven by clinical phenotype and PFCs. The protocol includes four aspects, namely network construction, module identification, the correlation between modules and features, and network visualization [28, 29]. Before analysis, the serum metabolic spectra (60 metabolites) were used as metabolite expression matrix, and clinical phenotype and PFCs of interest as trail matrix in the PT and CPP girls. Then the default WGCNA “step-by-step network construction” analysis was used to build modules. Firstly, the adjacencies between metabolites were calculated and constructed a topological overlap matrix (TOM). A hierarchical clustering tree was produced with the dissimilarity of the TOM and the modules were then selected by using the dynamic tree cut. Finally, the similar modules were merged by calculating the module eigenmetabolites (ME), clustering them and assigning a distance threshold (cut of 0.2). The detailed analysis process and network construction results could be found in section S2 and Figure S2 in the Additional file1.

## Results

### Demographic and clinical characteristics of the clinical cohort

According to the demographic and clinical characteristics (Table 1), the body mass index standard deviation score (BMISDS) of PP girls (0.48 ± 1.16) was significantly higher than that of both prepubertal (−0.32 ± 1.16) (*p* < 0.001) and adolescent girls (−0.42 ± 1.41) (*p* = 0.005), the BMISDS of CPP girls (0.70 ± 1.06) was significantly higher than that of both prepubertal (*p* < 0.001) and adolescent girls (*p* = 0.002), and the BMISDS of PT (0.33 ± 1.4) was also significantly higher than that of both prepubertal (*p* = 0.047) and adolescent (*p* = 0.0047). Obviously, the basal LH and FSH levels of PT girls (0.304 and 2.30 mIU/mL, respectively) were closer to prepubertal girls (0.256 and 2.46 mIU/mL, respectively), while those of CPP girls (2.73 and 5.01 mIU/mL, respectively) were closer to those of adolescent girls (2.93 and 5.85 mIU/mL, respectively), and estradiol and prolactin showed a similar trend. The serum levels of testosterone, dehydroepiandrosterone sulfate (DHEAS), free thyroxine (FT4), glucose, and urea/creatinine ratio were statistically significant between PT and CPP. The serum 25-hydroxyvitamin D (VD) level in CPP (61.86 nmol/L) was significantly lower than that of prepubertal (83.14 nmol/L) (*p* = 0.01) and PT (75.57 nmol/L) (*p* = 0.02).

**Table 1:** Demographic data and clinical characteristics of the study cohort

**Clinical phenotype**	**Prepubertal girls (*n*** **= 36)**	**PP girls (*n*** **= 146)**		**Adolescent girls (*n*** **= 28)**
Age at presentation (years)	6.1 ± 2.2	7.90 ± 1.4^a,b^		10.9 ± 1.3
Age of onset of complaints (years)	-	6.89 ± 0.73		-
BMI (kg/m^2^)	15.1 ± 1.60	16.4 ± 2.2 ^b^		16.3 ± 3.1
BMISDS	-0.32 ± 1.2	0.48 ± 1.2^a,b^		−0.42 ± 1.4
	**Prepubertal girls (*n* = 36)**	**PT girls (*n* = 40)**	**CPP girls (*n* = 30)**	**Adolescent girls (*n* = 28)**
Age at presentation (years)	6.1 ± 2.6	6.32 ± 1.0^a^	8.7 ± 0.65^a,b,c^	10.9 ± 1.3
Age of onset of complaints (years)	-	6.11 ± 0.56	7.32 ± 0.85	-
BMI (kg/m^2^)	15.1 ± 1.60	15.8 ± 2.2	17.0 ± 2.1^b,c^	16.3 ± 3.1
BMISDS	-0.32 ± 1.2	0.33 ± 1.4^a,b^	0.70 ± 1.1^a,b^	-0.42 ± 1.4
Basal LH (mIU/mL)	0.256 ± 0.16	0.304 ± 0.17^a^	2.73 ± 1.7^a,b,c^	2.93 ± 2.0
Basal FSH (mIU/mL)	2.46 ± 1.3	2.30 ± 0.96^a^	5.01 ± 2.9 ^a,b,c^	5.85 ± 3.1
GnRH stimulation test (mIU/mL)	-	-	LH peak (25.15 ± 18.0), LH/FSH (1.61 ± 0.89)	-
Estradiol (pg/mL)	20.00 ± 0.01	20.03 ± 7.61^a^	40.41 ± 28.6 ^b,c^	44.09 ± 18.9
Prolactin (ng/mL)	8.01 ± 2.6	6.59 ± 3.9^a^	10.79 ± 4.88 ^c^	10.99 ± 6.23
Testosterone (pg/mL)	-	< 0.10	0.20 ± 0.1 ^c^	-
DHEAS (μg/dL)	-	18.29 ± 13.1	75.75 ± 39.51^c^	-
VD (nmol/L)	83.14 ± 27.4	75.57 ± 14.5^a^	61.86 ± 21.3 ^b,c^	63.05 ± 13.8
FT4 (ng/dL)	0.98 ± 0.1	1.02 ± 0.14^a^	0.93 ± 0.09^c^	0.90 ± 0.1
TSH (Uiu/mL)	2.76 ± 1.5	2.18 ± 1.0	2.38 ± 1.2	2.35 ± 1.4
Glucose (mmol/L)	-	4.70 ± 0.38	5.05 ± 0.25^c^	-
Urea/creatinine	-	0.10 ± 0.02	0.08 ± 0.02^c^	-

*Abbreviations*: *PP* Precocious puberty, *PT* Premature thelarche, *CPP* Central precocious puberty, *BMI* Body mass index, *BMISDS* Body mass index standard deviation score, *LH* Luteinizing hormone, *FSH* Follicular-stimulating hormone, *DHEAS* Dehydroepiandrosterone sulfate, *VD* 25-hydroxyvitamin D, *FT4* Free thyroxine, *TSH* Thyroid-stimulating hormone

The data are expressed as the means ± standard deviation (S.D.). Student’s *t* test was used for comparisons between two groups. If the variance was homogeneous, the *p* value was calculated directly, otherwise Welch-Satterthwaite method was used:

^a^Significantly different compared with adolescent girls

^b^Significantly different compared with prepubertal girls

^c^Significantly different compared with PT

### Identifying the serum potential biomarkers of PP, CPP, and PT girls

**Fig. 2:**
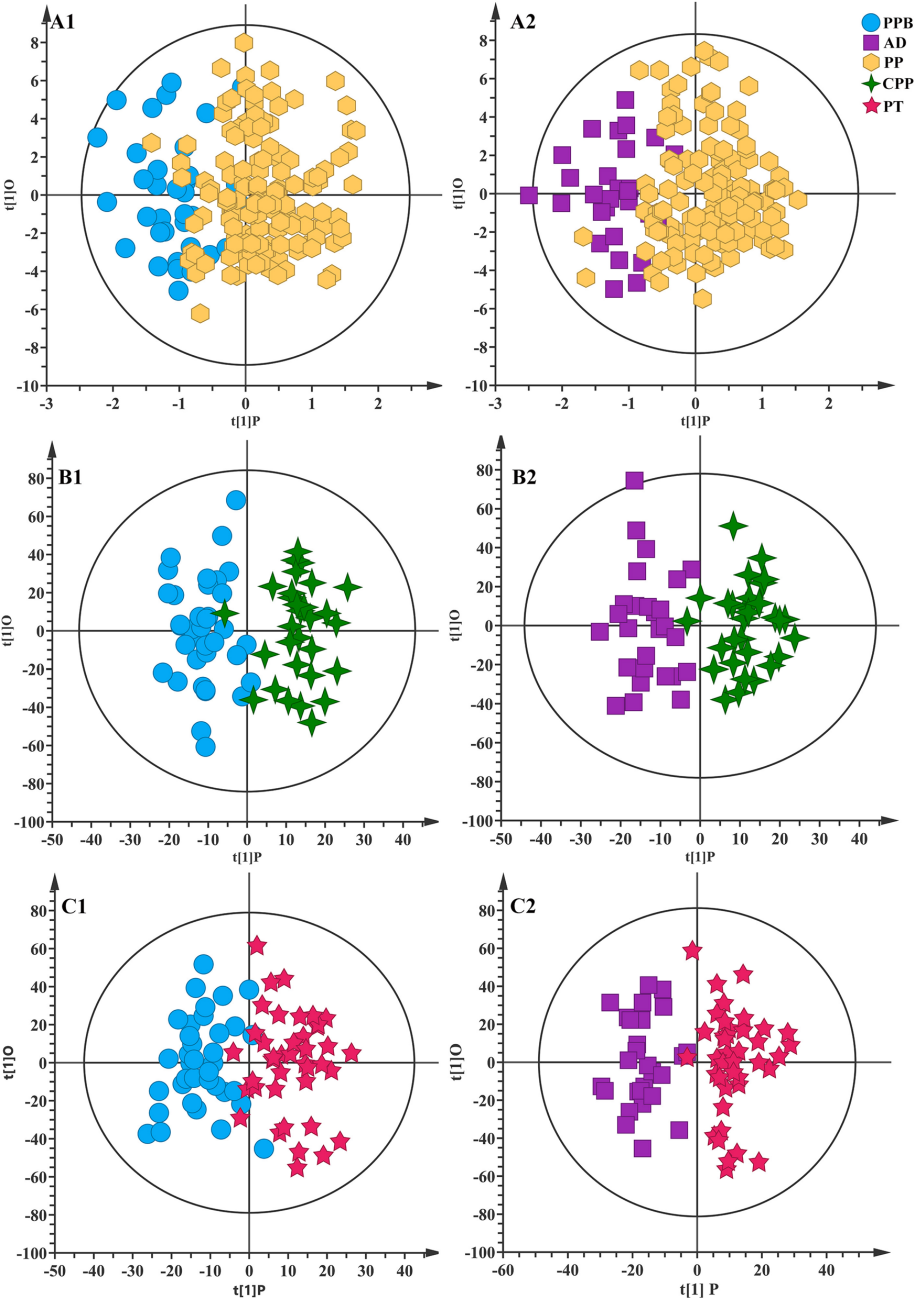
OPLS-DA score plots of serum samples. A1 The PP *vs.* prepubertal girls. A2 The PP *vs.* adolescent girls. B1 The CPP *vs.* prepubertal girls. B2 The CPP *vs.* adolescent girls. C1 The PT *vs.* prepubertal girls. C2 The PT *vs.* adolescent girls. The sample numbers of prepubertal, adolescent, PP, CPP, and PT girls were 36, 28, 146, 30, and 40, respectively. PPB: prepubertal; AD: adolescent

A total of 60 metabolites were identified from the serum ^1^H-NMR spectra of PP, prepubertal, and adolescent girls (Additional file 1: Figure S3 and Table S3). No obvious sample separation was observed between PP and prepubertal and adolescent girls in the PCA score plots of the NMR data (Additional file 1: Figures S4A and S4B). The OPLS-DA highlighted and maximized the metabolic differences between PP and prepubertal and adolescent as shown in Fig. 2A1 and A2. The favorable model parameters, including R^2^ for indicating the explained variances of the original data and Q^2^ for indicating the predictive ability of the model, revealed the obvious metabolic differences between PP with prepubertal and adolescent (Additional file 1: Table S4), and the results were further externally cross-validated by the permutation tests (Additional file 1: Figure S5 and Table S4). According to the screening criteria (VIP >1 and p-adj < 0.05), a total of 16 metabolites were selected as the potential biomarkers of PP girls when compared with prepubertal girls and adolescent girls as demonstrated in Additional file 1: Table S5.

To clarify the respective pathogenesis of CPP and PT and differentiate the two classifications from the serum metabolome, the serum metabolic profile of CPP or PT girls was compared with prepubertal and adolescent, respectively. The OPLS-DA model revealed the obvious metabolic differences between CPP and prepubertal or adolescent girls (Fig. 2B1, B2 and Additional file 1: Table S4) and between PT and prepubertal or adolescent girls (Fig. 2C1, C2 and Additional file 1: Table S4) though no obvious separation was observed in the corresponding PCA score plots (Additional file 1: Figures S4C&S4D). The results were confirmed by the permutation tests (Additional file 1: Figure S5 and Table S4). According to the screening criteria, 23 metabolites were screened out as the potential biomarkers of CPP when compared with prepubertal girls and adolescent girls, and 25 metabolites were screened out as the potential biomarkers of PT when compared with prepubertal girls and adolescent girls as demonstrated in Table 2. Among them, formate, ethanol, and 3-hydroxybutyrate were both significantly upregulated in CPP and PT (Table 2).

**Table 2:** Potential biomarkers in serum of CPP and PT

**Potential biomarker**	**Raw** *p* **value**	**VIP**	**FC**	**Age-adjusted**	
				**OR (95% C**I**)**	**p-adj**
**Central precocious puberty girls**					
Compared with the prepubertal girls					
Alanine	1.25E−02	2.250	0.909	0.408 (0.356 to 0.450)	2.01E−02
Creatine	2.00E−05	2.956	0.874	0.180 (0.167 to 0.193)	3.32E−02
Creatinine	3.65E−03	1.800	0.901	0.901 (0.069 to 0.084)	1.37E−02
Dihydrothymine	1.86E−03	2.364	0.916	0.916 (0.011 to 0.013)	1.46E−03
Glutamate	2.74E−02	1.592	0.863	0.863 (0.245 to 0.354)	3.12E−02
Glutamine	1.74E−02	2.328	1.080	1.082 (0.947 to 1.217)	7.89E−02
Histidine	2.05E−02	2.919	0.926	0.088 (0.080 to 0.096)	4.52E−02
Hypoxanthine	1.72E−03	1.537	0.440	0.049 (0.033 to 0.064)	2.09E−02
Isobutyrate	4.95E−03	2.458	0.895	0.037 (0.033 to 0.041)	1.95E−02
Isoleucine	1.93E−03	2.680	0.883	0.114 (0.102 to 0.126)	2.27E−03
Lactate	9.93E−03	1.965	0.794	3.121 (2.493 to 3.748)	1.72E−01
Lactose	1.00E−05	2.598	1.242	0.017 (0.013 to 0.020)	1.47E−02
Leucine	1.16E−02	2.811	0.941	0.146 (0.136 to 0.156)	1.55E−02
Methanol	1.88E−02	1.630	1.168	0.057 (0.040 to 0.073)	1.13E−01
Phenylalanine	4.66E−02	1.656	0.916	0.047 (0.041 to 0.052)	3.79E−02
Serine	8.15E−03	3.156	0.922	0.176 (0.160 to 0.192)	2.23E−02
Tyrosine	5.22E−03	2.020	0.858	0.062 (0.053 to 0.070)	2.17E−03
α-Glucose	9.58E−03	2.432	1.147	0.866 (0.660 to 1.071)	9.42E−03
β-Glucose	4.27E−02	2.258	1.124	1.021 (0.764 to 1.278)	3.64E−02
Compared with the adolescent girls					
3-Hydroxybutyrate	4.53E−03	2.436	1.184	0.090 (−0.021 to 0.191)	2.10E−02
Alanine	4.85E−03	2.274	0.869	0.406 (0.175 to 0.637)	1.39E−02
Creatinine	9.80E−04	2.572	0.892	0.106 (0.077 to 0.136)	6.30E−04
Ethanol	4.74E−02	2.061	1.112	0.111 (0.003 to 0.219)	4.42E−02
Formate	1.89E−02	1.802	1.240	0.006 (−0.002 to 0.015)	2.29E−02
Isoleucine	1.00E−05	3.187	0.873	0.128 (0.091 to 0.164)	3.10E−04
Methionine	3.97E−02	2.840	0.943	0.228 (0.175 to 0.281)	3.35E−03
Phenylalanine	3.22E−02	2.806	0.907	0.072 (0.048 to 0.096)	2.37E−03
Tyrosine	9.90E−04	3.383	0.862	0.087 (0.059 to 0.114)	4.70E−04
Uridine	6.26E−03	2.109	0.802	0.040 (0.023 to 0.056)	1.91E−03
Valine	4.61E−02	2.719	0.932	0.350 (0.233 to 0.467)	2.02E−02
α-Ketoisovalerate	1.61E−02	1.656	0.848	0.009 (−0.011 to 0.030)	9.97E−03
**Premature thelarche girls**					
Compared with the prepubertal girls					
Acetate	3.42E−02	1.510	1.091	0.059 (0.048 to 0.070)	3.61E−02
Asparagine	9.28E−03	2.527	1.085	0.567 (0.492 to 0.642)	5.93E−03
Dihydroxyacetone	2.79E−02	1.404	1.257	0.013 (0.005 to 0.022)	3.78E−02
Ethanolamine	1.30E−04	2.728	1.126	0.059 (0.050 to 0.068)	2.20E−04
Glycerol	4.65E−02	2.456	1.061	0.338 (0.294 to 0.382)	5.08E−02
Lipid	2.22E−02	1.615	0.943	1.928 (1.753 to 2.103)	3.23E−02
Methanol	1.86E−02	1.432	1.149	0.056 (0.039 to 0.074)	2.62E−02
*myo*−Inositol	4.65E−02	2.645	1.072	0.139 (0.119 to 0.159)	3.11E−02
*N*, *N*−Dimethylglycine	4.58E−02	1.429	1.104	0.013 (0.012 to 0.017)	3.98E−02
Ornithine	2.63E−02	1.795	1.103	0.117 (0.117 to 0.151)	1.76E−02
*para*-Hydroxybenzoate	1.81E−03	2.491	1.100	0.021(0.018 to 0.024)	2.05E−03
Trimethylamine N-oxide	2.61E−03	2.010	1.137	0.010 (0.008 to 0.012)	3.02E−03
Uridine	3.50E−04	2.655	1.348	0.009 (0.004 to 0.014)	2.90E−04
Very low-density lipoprotein	1.31E−02	1.747	0.814	4.077 (3.112 to 5.041)	1.90E−02
Compared with the adolescent girls					
1-Methylhistidine	3.44E−03	2.045	1.164	0.149 (−0.038 to 0.335)	1.20E−03
3-Hydroxybutyrate	4.40E−04	2.593	1.344	0.109 (0.079 to 0.139)	4.04E−02
Acetate	9.00E−05	2.261	1.177	0.007 (−0.008 to 0.021)	9.00E−05
Acetoacetate	2.55E−03	2.053	1.187	0.436 (0.282 to 0.590)	6.13E−03
Acetone	3.15E−02	1.778	1.254	0.395 (0.196 to 0.593)	1.50E−01
Asparagine	1.59E−02	2.494	1.076	0.119 (0.077 to 0.162)	2.32E−01
Choline	5.80E−04	2.185	1.151	2.984 (−0.763 to 6.731)	6.88E−01
Creatine	3.00E−05	2.061	1.151	1.606 (0.950 to 2.263)	1.00E−05
Dihydroxyacetone	1.73E−02	1.210	1.295	0.047 (0.030 to 0.064)	3.29E−01
Ethanol	3.01E−03	1.969	1.251	0.036 (0.026 to 0.046)	3.13E−02
Ethanolamine	5.70E−03	2.184	1.090	0.163 (0.100 to 0.227)	3.50E−04
Formate	5.69E−03	1.389	1.478	0.025 (0.013 to 0.036)	1.99E−02
Glycerol	2.15E−02	2.126	1.072	0.727 (−2.896 to 4.350)	2.75E−02
Glycine	2.38E−02	1.898	1.121	0.016 (−0.004 to 0.037)	2.13E−02
Histidine	4.14E−02	2.572	1.081	0.149 (−0.038 to 0.335)	1.12E−02
Lactate	4.25E−02	1.819	1.260	0.109 (0.079 to 0.139)	9.49E−02
Lipid	3.80E−04	1.867	0.907	0.007 (−0.008 to 0.021)	8.20E−04
*N*, *N*-Dimethylglycine	2.57E−02	1.820	1.085	0.436 (0.282 to 0.590)	1.05E−02
*para*-Hydroxybenzoate	9.02E−03	2.077	1.089	0.395 (0.196 to 0.593)	1.53E−01
Serine	1.00E−05	2.281	1.148	0.119 (0.077 to 0.162)	3.00E−05
Succinate	1.34E−02	2.344	1.189	2.984 (−0.763 to 6.731)	2.30E−04
Very low-density lipoprotein	2.15E−03	1.684	0.761	1.606 (0.950 to 2.263)	1.27E−03
α-Ketoisovalerate	1.34E−02	1.323	0.853	0.047(0.030 to 0.064)	2.32E−02

Raw *p* value, calculated by Student’s *t* test, *p* < 0.05 regard as significantly changed

FC, fold change of metabolite, FC = C disease/C control, where FC > 1 means elevated content and FC < 1 indicates decreased content of metabolite

*VIP* Variable importance for projection, *OR* Odds ratio

p-adj, after age correction of raw *p* value performed by linear regression analysis

### Random forest modeling validation of serum-specific biomarkers of CPP and PT girls

**Fig. 3:**
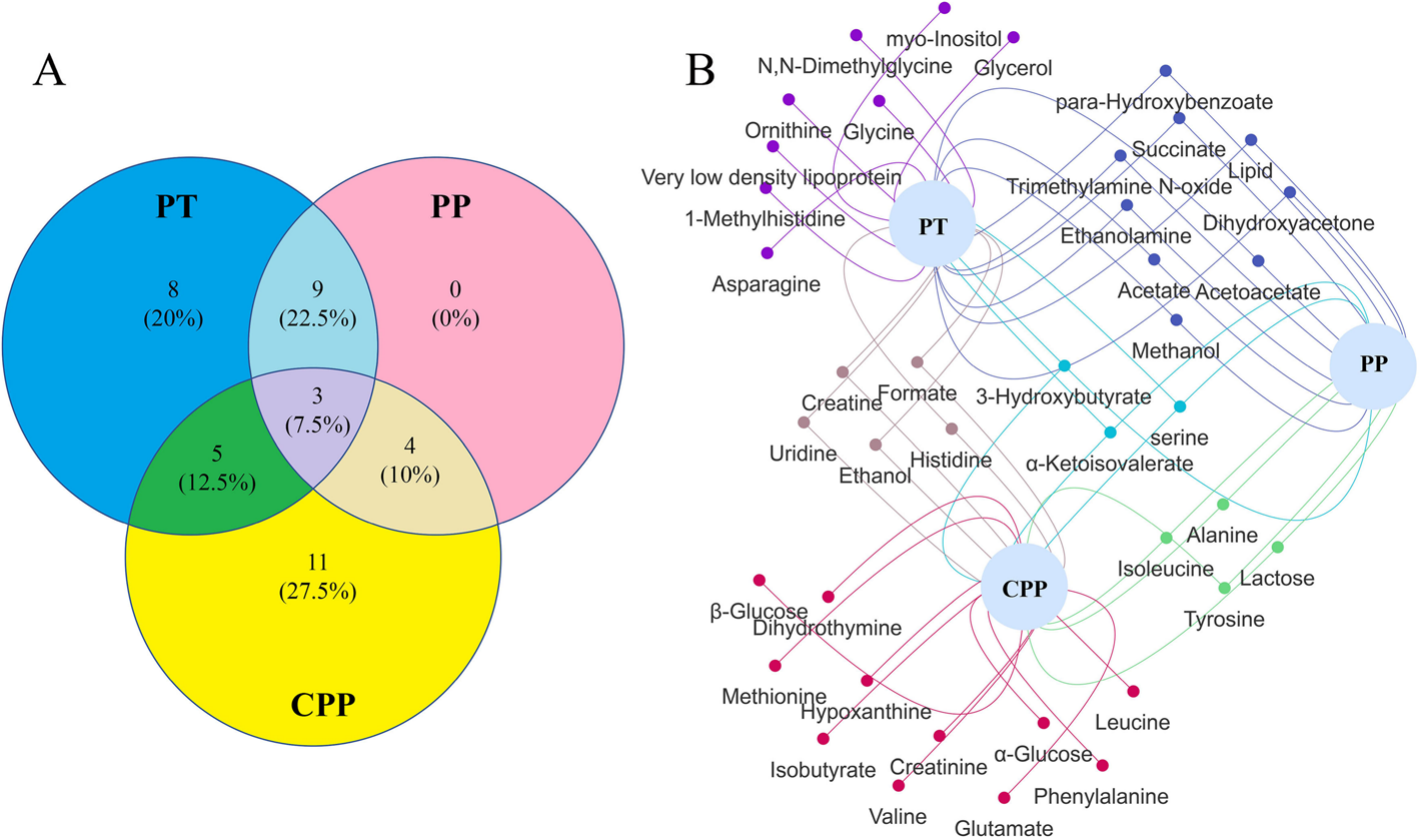
The potential biomarkers of PP, PT, and CPP.** A** The Venn plots of the potential biomarkers in the PP, PT, and CPP. **B** The bipartite graph of the potential biomarkers in the PP, PT, and CPP

**Fig. 4:**
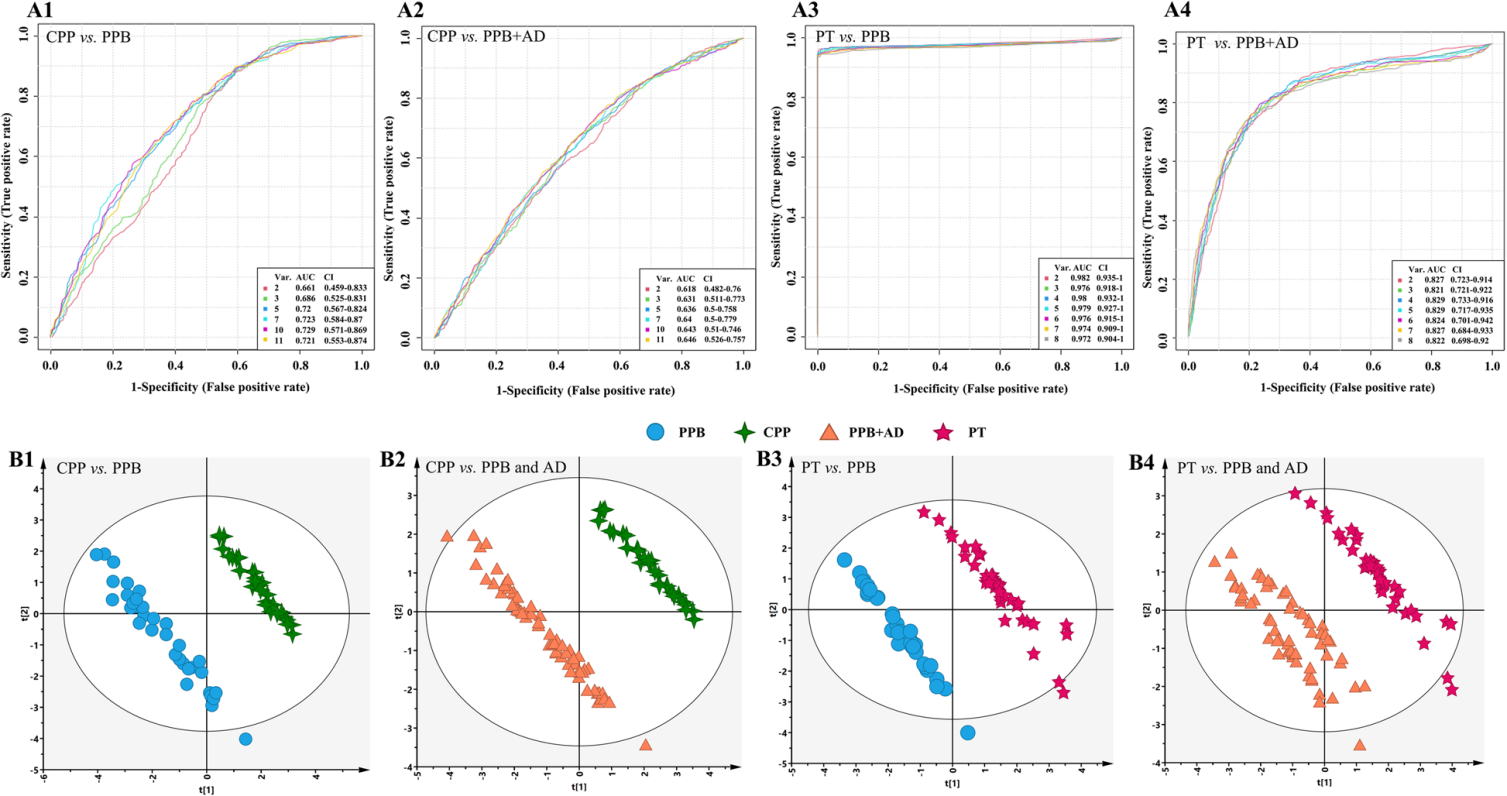
An exploratory ROC curve analysis (**A**) and the validation PLS-DA models (**B**) based on the specific biomarkers. ROC curve analysis for the predictive power of specific biomarkers of CPP for distinguishing CPP from prepubertal girls (A1) and for distinguishing CPP from prepubertal integrated adolescent girls (A2). ROC curve analysis for the predictive power of specific biomarkers of PT for distinguishing PT from prepubertal girls (A3) and for distinguishing PT from prepubertal integrated adolescent girls (A4). The validation models were constructed to classify between CPP and prepubertal girls (B1), between CPP and prepubertal integrated adolescent girls (B2), between PT and prepubertal girls (B3), between PT and prepubertal integrated adolescent girls (B4). The sample numbers of prepubertal, adolescent, CPP and PT girls were 36, 28, 30, and 40, respectively. PPB: prepubertal; AD: adolescent

The potential biomarkers of PP, CPP, and PT girls partially overlap (Fig. 3A), which is understandable because of their similar metabolic characteristics. A comparative analysis was performed to get their individual specific biomarkers. The results indicated that PP shared the potential biomarkers with CPP or PT, and eleven potential biomarkers, including glutamine, α-&β-glucose, dihydrothymine, methionine, hypoxanthine, isobutyrate, creatinine, valine, leucine, and phenylalanine, are specified to CPP, and eight potential biomarkers, including 1-methylhistidine, *myo*-inositol, *N,N*-dimethylglycine, very low-density lipoprotein (VLDL), glycerol, ornithine, asparagine, and glycine, are specified to PT (Fig. 3B). An exploratory ROC curve analysis based on random forest was used to evaluate the sensitivity and specificity of the specific biomarkers of the diseases (Fig. 4A). The AUC between CPP and prepubertal girls varied depending on the combinations of the eleven specific biomarkers, ranging from 0.66 to 0.73, and reached 0.721 (95% CI 0.553–0.874) when the eleven specific biomarkers were integrated (Fig. 4A1), while the AUC between CPP and prepubertal integrated adolescent girls was 0.646 (95% CI 0.526-0.757) at the combination of the eleven specific biomarkers (Fig. 4A2). The specific biomarkers for PT exhibit higher sensitivity. The AUC between PT and prepubertal girls was 0.97–0.98 and reached 0.972 (95% CI 0.904–1) at the combination of the eight specific biomarkers (Fig. 4A3). And it reached 0.822 (95% CI 0.698–0.92) between PT and prepubertal integrated adolescent girls when the eight specific biomarkers applied (Fig. 4A4). These results indicated the favorable sensitivity and specificity of the specific biomarkers of CPP and PT.

To further verify the superiority of disease-specific biomarkers in classification to the healthy girls, the PLS-DA models were reconstructed with the specific biomarkers as variables (Fig. 4B and Additional file 1: Table S4). The score plot showed a clear distinction between CPP and prepubertal girls (Fig. 4B1), and CPP could be clearly distinguished from prepubertal and adolescent girls with excellent predictive and explanatory power (Fig. 4B2 and Additional file 1: Table S4). A similar result was obtained in PT (Fig. 4B3, B4 and Additional file 1: Table S4).

### The disturbed metabolic pathway and network induced by CPP and PT

**Fig. 5:**
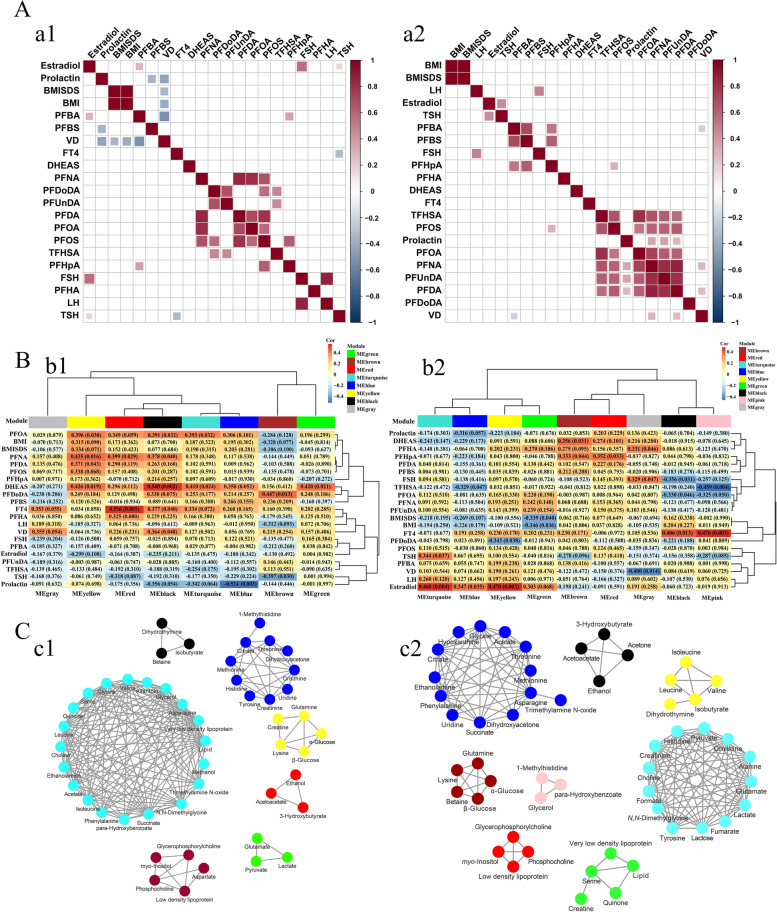
The core metabolic network of CPP (**A**) and PT (**B**). (HPG: hypothalamic-pituitary-gonadal; HPA: hypothalamic-pituitary-adrenal; GnRH: gonadotropin-releasing hormone; CRH: corticotropin releasing hormone; ACTH: adrenocorticotropic hormone; LH: luteinizing hormone; FSH: follicular-stimulating hormone; DHEAS: dehydroepiandrosterone sulfate; VLDL: very low-density lipoprotein.)

To gain insight into the metabolic disorders of CPP and PT, the metabolic pathways were enriched based on the potential biomarkers through online database MetaboAnalyst 5.0, and the impact value was used to evaluate the crucial pathways involved in the occurrence of diseases. Based on the criterion of pathway impact > 0.1 and *p* < 0.05, six disturbed metabolic pathways were screened out from CPP, including aminoacyl-tRNA biosynthesis, valine, leucine, and isoleucine biosynthesis, phenylalanine, tyrosine and tryptophan biosynthesis, phenylalanine metabolism, butanoate metabolism, and histidine metabolism (Additional file 1: Figure S6A), while seven metabolic pathways were significantly disordered in PT, including butanoate metabolism, synthesis and degradation of ketone bodies, glyoxylate and dicarboxylate metabolism, glycine, serine, and threonine metabolism, glycerolipid metabolism, histidine metabolism, and aminoacyl-tRNA biosynthesis (Additional file 1: Figure S6B). Furthermore, to better understand the process of diseases, the core metabolic network of CPP and PT were constructed to explain their individual pathogenesis based on the potential biomarkers via integrating the database of KEGG and HMDB. The links between hypothalamic-pituitary-gonadal-adrenal (HPGA) axis initiation and metabolism (including phenylalanine, tyrosine, and tryptophan biosynthesis, glycine, serine and threonine metabolism, glycolysis/gluconeogenesis, alanine, aspartate and glutamate metabolism, pyrimidine metabolism, aminoacyl-tRNA biosynthesis, and pyruvate metabolism and butanoate metabolism) were mainly shown in the core metabolic network of CPP (Fig. 5A). Metabolic pathway interconnections including glycerolipid metabolism, galactose metabolism, amino acid metabolism, pyruvate metabolism, butanoate metabolism, and pyrimidine metabolism were mainly shown in the core metabolic network of PT (Fig. 5B).

### Intra-group association between PFCs and clinical phenotypes in the CPP and PT girls

To understand the latent effects of PFCs on the occurrence of PP, serum PFCs in the PT and CPP girls were analyzed. The detailed detection results of PFCs are shown in Additional file 1: Table S6, which show the effectiveness of the detection method and the stability of the instrument. The levels of PFHPA and PFHA were statistically different between CPP and PT groups (*p* < 0.05), and the other nine PFCs were no statistical difference (Additional file 1: Table S7).

**Fig. 6:**
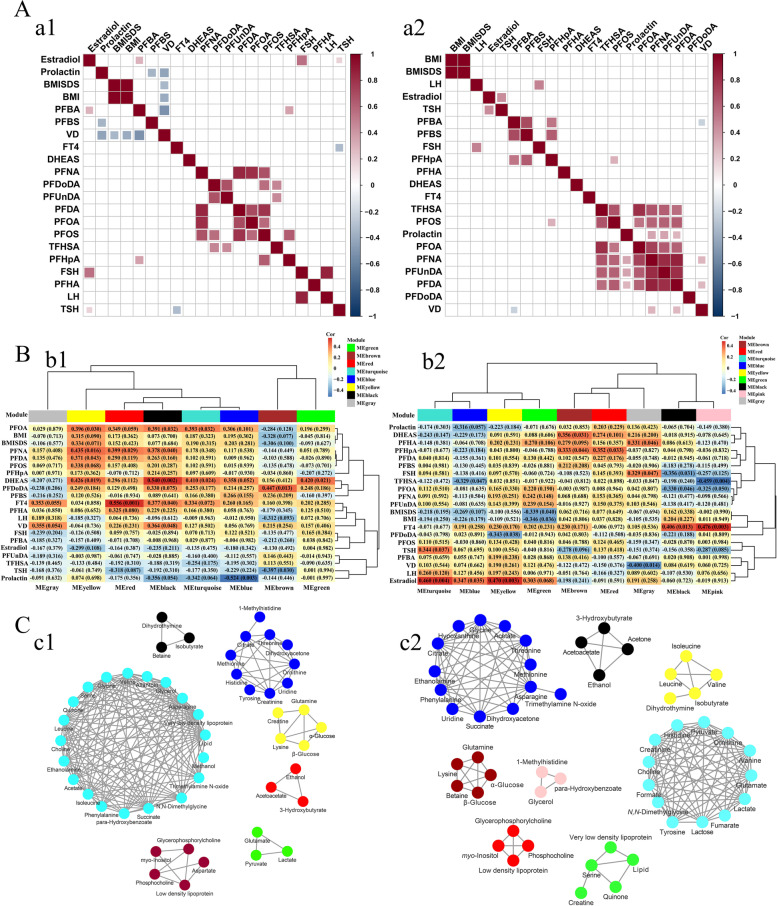
Visualization plot of the association of clinical phenotype-PFCs-metabolic characteristics. **A** Spearman correlation analysis of PFCs and clinical phenotypes in the CPP (a1) and PT (a2) girls, red and blue represent positive and negative correlation, respectively (noted: only correlation values that meet *p* < 0.05 are presented on the heat map). **B** Module-trait associations of CPP (b1) and PT (b2), where each row corresponds to a module eigenmetabolite, column to a trait, respectively. Each cell contains the corresponding correlation and *p* value. **C** Visualization of module metabolites of CPP (c1) and PT (c2)

Spearman correlation analysis showed strong positive correlations among PFCs in the CPP girls. Estradiol was positively correlated with PFBA, TSH, and FSH. Prolactin was negatively correlated with PFBS and VD (Fig. 6a1 and Additional file 2: Table S8). In PT, the strong positive associations were also observed between PFCs, and prolactin was positively correlated with PFDUnDA, PFDA, and PFNA. VD was positively correlated with PFDA and PFNA, negatively correlated with PFBA (Fig. 6a2 and Additional file 2: Table S9).

### Determination the trait-driven metabolite modules in the CPP and PT girls

WGCNA is a systems biological method for understanding the correlated patterns between variables across different samples and has been widely used to find clusters or modules of metabolites. In this study, the metabolites were finally divided into eight and nine modules by WGCNA in CPP and PT, respectively, and the different modules were represented by different colors (the metabolites that do not belong to any module are classified as gray modules) (Additional file 1: Figure S2B). Furthermore, module-to-module correlation and cluster analysis are shown in Additional file 1: Figure S2C.

In module-trait heatmap, the driver module was determined based on the criterion |cor| > 0.30 and *p* < 0.05. In CPP, PFOA, PFNA, and PFDA mainly drive MEyellow, PFDoDA mainly drives MEbrown, DHEAS and VD mainly drive MEblack, FT4 mainly drives MEred, LH and FSH mainly drive MEbrown, and prolactin mainly drives MEblue (Fig. 6b1). In PT, PFOA mainly drives MEblack, TFHSA mainly drives MEpink, PFDoDA mainly drives MEyellow, and PFHpA mainly drives MEred, DHEAS mainly drives MEbrown, FSH mainly drives MEblack, BMISDS and body mass index (BMI) mainly drives MEgreen, FT4 mainly drives MEpink, TSH mainly drives MEturquoise, and estradiol mainly drives MEyellow (Fig. 6b2). The metabolites in each module are shown in Fig. 6C.

## Discussion

### PFCs contribute to PP by affecting endocrine disorders in girls

In this study, the levels of basal LH and FSH, estradiol, prolactin, testosterone, and DHEAS in PT and CPP indicated that the HPG axis is not activated in the PT girls but activated in the CPP girls, which was also confirmed by the GnRH stimulation test [30]. With activation of HPG axis, hypothalamus increasingly secretes gonadotropin GnRH, anterior pituitary gland increasingly secretes FSH and LH, and gonad increasingly secretes estradiol and testosterone. The higher level of estradiol (aromatized from testosterone) in girls before puberty is associated with an earlier thelarche, and it can affect and maintain cognitive function, regulate the sexual behavior and ovulation in the brain, and regulate higher order neural function [31, 32]. Behr et al. found that PFOA and PFOS can enhance estradiol-stimulated estrogen receptor β activity, and PFOS and PFBA can enhance dihydrotestosterone-stimulated androgen receptor activity [33]. In this study, PFBA was positively correlated with estradiol, PFBS was negatively correlated with prolactin in the CPP girls, and PFUnDA, PFDA, and PFNA were positively correlated with prolactin in the PT girls, which indicated that PFCs mainly caused developmental and reproductive toxicity by disrupting the body’s endocrine homeostasis, thus leading to an imbalance in the body’s steroid hormone secretion.

DHEAS is a stable marker for adrenal androgenic activity. At the biological level, DHEAS has effects on brain development, sexuality, mood and cognition, cardiovascular disease, stroke, and mortality [34]. Relevant longitudinal studies show that higher DHEAS level at the age of 8 predicts early menarche and doubles the risk of pubic hair development in girls [35]. In this study, the higher level of DHEAS in the CPP girls may be the result of a combination of adrenaline secretion and gonadal secretion, and it further leads to significant secondary sexual characteristics of the CPP girls. On the other hand, the increased level of DHEAS may potentially provide additional energy for the metabolic costs of early brain development, and also acts as a co-factor in promoting cortical maturation, thus leading to increased capacity for mentalizing and perspective-taking before the onset of reproductive maturation. This conclusion could be supported by the WGCNA analysis, where MEyellow in CPP and MEbrown in PT driven by DHEAS showed glucose aggregation. In addition, the significantly higher level of glucose in the CPP girls than in the PT girls also indicated a more active energy metabolism in CPP.

Interestingly, in this study, the BMI of CPP was higher than that of other groups. Studies have shown that the HPG axis initiates when girls’ body reaches a certain fat and/or protein mass [36]; therefore, the occurrence of CPP may be closely related to high BMI of girls. It is possible that obesity or overweight can promote the occurrence and development of CPP, which could be confirmed by the findings of many other scholars [37, 38]. In addition, the relationship between PFCs and VD in CPP and PT indicated PFCs partly disturbed osteogenesis [39], and also revealed new potential long-term PFC impacts on children.

### PFCs mediate perturbation of the core metabolic network of the CPP girls

CPP is primarily an early initiation of the HPGA axis, and hypothalamus generates a GnRH pulse that stimulates the pituitary gonadotropin secretion. Therefore, the corresponding core metabolic network mainly reflects the cause of activation of the HPGA axis and the metabolic disorder in the CPP girls. The link of clinical phenotype-PFCs-metabolic characteristics in the CPP girls indicated that PFCs may cause disturbance of metabolic network of CPP girls by disturbing endocrine homeostasis and/or directly affecting metabolite characteristics.

Tyrosine and phenylalanine are the precursor for catecholamines including tyramine, dopamine, epinephrine, and norepinephrine. The sympathetic nervous system neurotransmitters and catecholamines, especially norepinephrine, play an important role in the regulation of GnRH neurons [40]. The downregulated levels of phenylalanine and tyrosine in serum of the CPP girls indicated that the biosynthesis of phenylalanine, tyrosine, and tryptophan was disturbed, which keeps consistent with the previous metabolomics results [5]. The downregulation of phenylalanine and tyrosine levels may be due to the more consumption of norepinephrine for the activation of HPG axis, resulting in lower levels of its precursor substances. Such conclusion could be confirmed by the WGCNA analysis, where the modules MEblue and MEturquoise were primarily driven by prolactin and DHEAS (Fig. 6b1, c1).

Serine and glycine are connected through biosynthesis to provide necessary synthetic precursors of proteins, nucleic acids, and lipids. At the same time, serine homeostasis plays a vital role in maintaining brain energy metabolism [41]. The homeostasis of serine/glycine is essential for the proliferation of human primary muscle progenitor cells and efficient skeletal muscle regeneration [42]. Therefore, the decreased serine level in the CPP girls may be one of the reasons for the lifelong high short stature in adults, but further research is needed to confirm it. Creatine is essential in maintaining human growth, development, and health and can improve skin and bone health [43]. Creatinine was selected as a biomarker for predict CPP in Qi’s study [5], and it was regarded as one of the specific biomarkers for CPP in this study. Creatinine is linked to muscle mass, and the lower level of serum creatinine observed in the CPP girls could be a result of the excessive weight and relative low muscle/fat ratio caused by low physical activity. In the MEyellow and MEblue, creatine and creatinine are mainly related to amino acids and driven by multiple PFCs (PFOA, PFNA, PFDA), DHEAS, and prolactin (Fig. 6b1, c1), indicated that exposure to PFCs and/or endocrine disturbance will cause amino acid metabolism disturbance, thus affecting the bone growth of girls.

The increased levels of α-&β-glucose and lactose and decreased level of hypoxanthine in the CPP girls suggested the disturbed glycolysis/gluconeogenesis (energy metabolism). PFOA, PFDA, and PFNA were positively correlated with PFOS and mainly drove MEyellow containing α-&β-glucose. Studies have shown that the accumulation of PFCs, especially PFOS, contributes to the disorder of lipid and glucose metabolisms in children, but the mechanism is still in the initial stage of exploration [12, 44]. Compared with adolescent girls, the CPP girls had lower insulin sensitivity, glucose and lipid metabolism profile, and body composition, and the metabolic disturbance remained unchanged even after 1 year of GnRH treatment [45, 46]. The accumulation of PFCs may be a crucial reason for the decreased insulin sensitivity and high level of serum glucose in the CPP girls [47].

Glutamate and GABA are the principal excitatory and inhibitory neurotransmitters. Their interactions with GnRH neurons, including the regulation of GnRH gene and protein expression, hormone release, and modulation by estrogen, are critical to age-appropriate changes in reproductive function [48]. Glutamate is also crucial for bone growth and reconstruction [49]. In our study, the significantly decreased glutamate level in the CPP girls implies its dynamic change in the development of CPP. We speculated that the increased glutamate level stimulated the HPG axis and induced CPP in the early stage. However, the occurrence of CPP is often accompanied with rapid height growth, and subsequently the glutamate in bone and blood is excessively consumed at a certain stage of CPP.

In addition, the downregulated levels of isoleucine, alanine, valine, methionine, histidine, and α-ketoisovalerate in the CPP girls indicated the disturbed aminoacyl-tRNA biosynthesis. The disruption of aminoacyl-tRNA biosynthesis further supports our inference that body needs to consume large amounts of amino acids during the initial growth spurt and rapid bone maturation stage of the CPP girls. The significantly reduced level of VD in the CPP girls further indicated the affected bone growth of the CPP girls. It is possible that the lack of VD affected the absorption and deposition of calcium in the bone, thus affecting the health and growth of bones. Balance of aminoacyl-tRNA biosynthesis is closely related to bone health, and its disruption can lead to osteocyte protein synthesis dysfunction, marrow hypoplasia, and osteoporosis [50]. This may also be the reason for the short height of CPP girls at adult. However, whether appropriate supplementation of amino acids in diet can improve the adult height, or whether aminoacyl-tRNA biosynthesis can become a targeted therapeutic metabolic pathway for CPP girls is worthy of further research and discussion.

Purines and pyrimidines give prominent contributions in the development of the central nervous system, but the exact molecular mechanisms remain unclear [51]. PP girls have an increased risk of precocious sexual behavior and an increased prevalence of mental disorders such as depression and anxiety in adulthood [52, 53]. In this study, the downregulated serum levels of hypoxanthine, dihydrothymine, and uridine were observed in the CPP girls. Dihydrothymine belongs to MEblack jointly driven by PFOA, PFNA, DHEAS, FT4, and VD. This suggested that the disturbed pyrimidine metabolic pathway may be caused by the joint action of external environment and internal factors. CPP girls need to advance adaption to the cognitive, emotional, and changes of puberty prematurely, therefore, the sense of anxiety and depression increases significantly, resulting in a certain disorder of pyrimidine metabolism.

### PFCs mediate perturbation of the core metabolic network of the PT girls

PT does not involve the early initiation of HPGA axis and keep normal growth of height and maturation of bone age. Its occurrence is mainly due to the breast enlargement caused by exogenous hormone intake. In Qi et al. study [5], upregulated levels of succinate and 1-methylhistidine in urine can effectively predict PT. In this study, it was found that most amino acids showed upregulated in PT girls, including the specific biomarker 1-methylhistidine and the potential biomarker succinate. Amino acids are not only the building blocks of proteins and an indispensable component of cells, but also play versatile roles in regulating cell metabolism, proliferation, differentiation, and growth by themselves or their derivatives. Their requirements vary at the various stages of children’s growth and development [54, 55]. Studies have shown that arginine and ornithine supplementation can promote the secretion of growth hormone and insulin growth factor-1 [56]. We speculated that the nutritional diets promote the high level of amino acid in the PT girls for providing energy for the growth and development. But the more relevant reason may be that exposure to PFCs disturbs the body’s amino acid metabolism, endocrine homeostasis, and vitamin level, which will affect the girl’s bone health and growth.

In this study, the metabolites in glycerolipid metabolic pathway and galactose metabolic pathway including *N,N*-dimethylglycine, ethanolamine, glycerol, dihydrothymine, and *myo*-inositol were upregulated in the PT girls. Studies have shown that thyroid dysfunction will affect the body’s glycerol metabolism and gluconeogenesis pathway [57, 58]. In this study, FT4 and TFHSA mainly drive MEpink containing glycerol, and PFHpA mainly drives MEred containing low-density lipoprotein, *myo*-inositol, phosphocholine, and glycerophosphorycholine, suggesting that the PFCs exposure and high level FT4 may be the main reason contributed to the disorder of serum glycerolipid metabolic and galactose metabolism in PT. However, this study cannot directly prove the causation between the elevated FT4 level and PFCs exposure in PT.

Interestingly, the disordered trend of pyruvate metabolism and butyrate metabolism kept almost consistent between CPP and PT (Fig. 5), indicating that pyruvate metabolism and butyrate metabolism disorder are possibly a common phenomenon of PP. In other words, simultaneously elevated levels in serum formate, ethanol, and 3-hydroxybutyrate may serve as the early diagnostic indicators for CPP and PT, i.e., precocious puberty in girls, but the stratification of PP still needs to be further determined based on the serum-specific biomarkers. Ethanol may act on bone remodeling including osteocyte apoptosis [59, 60]. Elevated level of 3-hydroxybutyrate will inhibit the differentiation and growth of (pre)-chondrocytes [61] and participate in the process of osteoporosis [62]. There is a strong correlation between PFOA and PFNA in CPP and PT. Ethanol and 3-hydroxybutyrate belong to MEred which were jointly driven by PFNA and FT4 in CPP. In PT, ethanol and 3-hydroxybutyrate belong to the MEblack which were jointly driven by PFOA and FT4. It is suggested that PFC exposure and/or fluctuations in FT4 level will lead to elevated ethanol and 3-hydroxybutyrate in PP and thus affects bone growth and development, but more evidence is needed to prove this point.

It should be pointed out that this study had several limitations. Firstly, the results were obtained from a smaller-sample-size cohort, and measurements in an expanded cohort are necessary to validate the disease-specific biomarkers and determine their universality. Secondly, clinical reports have revealed the gender-related differences of PP not only in symptoms but also in pathophysiology. Therefore, the present findings could not naturally extend to boys, and further study on the sex-specific metabolic characteristics of PP is needed to comprehensively understand the underlying pathogenic mechanisms of PP. Finally, the detailed mechanism of how PFCs lead to endocrine homeostasis imbalance still keeps unclear and needs further study.

## Conclusions

In summary, based on the cross-metabolomics analyses, our study showed that formate, ethanol, and 3-hydroxybutyrate may serve as the early diagnostic indicators for the CPP and PT, i.e., precocious puberty in girls, and eleven CPP-specific biomarkers and eight PT-specific biomarkers in serum exhibited good sensitivity, which can facilitate the classification diagnosis of CPP and PT girls. The link of clinical phenotype-PFCs-metabolic characteristics in CPP and PT by WGCNA method revealed that PFC exposure is associated with endocrine homeostasis imbalance in the CPP and PT girls, and thus directly or indirectly drives metabolic changes and forms perturbations of the overall metabolic network. These findings will provide a potential diagnostic and stratification approach for the clinical diagnosis of precocious puberty in girls, and also raise social awareness that reducing exposure to PFC compounds may be an important strategy for preventing the occurrence and development of precocious puberty in girls.

### Supplementary Information

**Additional file 1:** **Table S1.** LC-MS/MS analytical mobility elution procedure. **Table S2.** Retention time, transitions and MS/MS conditions of the analytes. **Table S3.** The identified metabolites from ^1^H-NMR spectra of the girls’ serum samples. **Table S4.** Summary of the quality parameters of the multivariate statistical analysis. **Table S5.** The potential biomarkers in serum of PP. **Table S6.** Linear range, correlation coefficient, LOD and recovery of PFCs (*n* = 5). **Table S7.** Concentration and statistical analysis of serum PFCs in the CPP and PT groups. **Figure S1.** Sample preparation and detection procedure of serum PFCs. **Figure S2.** WGCNA related visualization diagram. **Figure S3.** Mean ^1^H-NMR spectra of serum from prepubertal, PP, PT, CPP and adolescent girls. **Figure S4.** PCA score plots of serum samples. **Figure S5.** Permutation test analysis to test the over-fitting of OPLS-DA model. **Figure S6.** The pathways enrichment analysis of the CPP (A) and PT (B) base on the potential biomarkers via MetaboAnalyst 5.0.

**Additional file 2:** **Table S8.** Spearman correlation analysis between clinical phenotype and PFCs in CPP girls.** Table S9.** Spearman correlation analysis between clinical phenotype and PFCs in PT girls.

## Data Availability

The datasets used and/or analyzed during the current study are available from the corresponding author on reasonable request.

## Abbreviations

^1^H-NMR: One-dimensional nuclear magnetic resonance hydrogen spectrum
AUC: Area under ROC curve
BMI: Body mass index
BMISDS: Body mass index standard deviation score
Cl: Confidence interval
DHEAS: Dehydroepiandrosterone sulfate
FSH: Follicular-stimulating hormone
FT4: Free thyroxine
GnRH: Gonadotropin-releasing hormone
HMDB: Human Metabolome Database
HPG: Hypothalamic-pituitary-gonadal
HPGA: Hypothalamic-pituitary-gonadal-adrenal
KEGG: Kyoto Encyclopedia of Genes and Genomes
LH: Luteinizing hormone
ME: Module eigenmetabolites
NMR: Nuclear magnetic resonance
OPLS-DA: Orthogonal partial least squares-discriminant analysis
p-adj: *p* value after age correction
PCA: Principal component analysis
PFBA: Perfluoro-n-butanoic acid
PFBS: Potassium perfluoro-1-butanesulfonate
PFCs: Perfluorinated compounds
PFDA: Perfluoro-n-decanoic acid
PFDoDA: Perfluoro-n-dodecanoic acid
PFHA: Perfluoro-n-hexanoic acid
PFHpA: Perfluoro-n-heptanoic acid
PFNA: Perfluoro-n-nonanoic acid
PFOA: Perfluoro-n-octanoic acid
PFOS: Potassium perfluoro-1-octanesulfonate
PFUnDA: Perfluoro-n-undecanoic acid
ROC: Receiver operating characteristic
TFHSA: Potassium perfluoro-1-hexanesulfonate
TOM: Topological overlap matrix
TSH: Thyroid-stimulating hormone
VD: 25-hydroxyvitamin D
VIP: Variable importance for projection
VLDL: Very low-density lipoprotein
WGCNA: Weighted gene co-expression network analysis
