# Supplementary material for: Metabolic characteristics and pathogenesis of precocious puberty in girls: the role of perfluorinated compounds
Source: BMC Med. 2023 Aug 25;21:323. doi: 10.1186/s12916-023-03032-0 (PMC10463894; doi:10.1186/s12916-023-03032-0)
Supplement: Supplementary file 1 — Additional file 1: Table S1. LC-MS/MS analytical mobility elution procedure. Table S2. Retention time, transitions and MS/MS conditions of the analytes. Table S3. The identified metabolites from 1H-NMR spectra of the girls’ serum samples. Table S4. Summary of the quality parameters of the multivariate statistical analysis. Table S5. The potential biomarkers in serum of PP. Table S6. Linear range, correlation coefficient, LOD and recovery of PFCs (n = 5). Table S7. Concentration and statistical analysis of serum PFCs in the CPP and PT groups. Figure S1. Sample preparation and detection procedure of serum PFCs. Figure S2. WGCNA related visualization diagram. Figure S3. Mean 1H-NMR spectra of serum from prepubertal, PP, PT, CPP and adolescent girls. Figure S4. PCA score plots of serum samples. Figure S5. Permutation test analysis to test the over-fitting of OPLS-DA model. Figure S6. The pathways enrichment analysis of the CPP (A) and PT (B) base on the potential biomarkers via MetaboAnalyst 5.0. [file 12916_2023_3032_MOESM1_ESM.docx]

Supplementary material

S1 Detection of serum perfluorinated compounds (PFCs)

Abbreviations:

PFOA: Perfluoro-n-octanoic acid; PFOS: Potassium perfluoro-1-octanesulfonate;

PFBA: Perfluoro-n-butanoic acid; PFUnDA: Perfluoro-n-undecanoic acid;

PFDoDA: Perfluoro-n-dodecanoic acid;

PFBS: Potassium perfluoro-1-butanesulfonate;

PFDA: Perfluoro-n-decanoic acid; PFHpA: Perfluoro-n-heptanoic acid;

PFHA: Perfluoro-n-hexanoic acid; TFHSA: Potassium perfluoro-1-hexanesulfonate;

PFNA: Perfluoro-n-nonanoic acid; MTBE: Methyl-tert-buty ether;

TBA: Tetra-n-butylammonium hydrogen sulfate

S1.1 Materials and methods

PFOA, PFOS, TFHSA, PFHA, PFBA, PFBS, PFDoDA, PFUnDA, PFNA and PFDA were purchased from AccuStandard (New Haven, CT, USA); PFOA ^13^C_8_ was purchased from Cambridge Isotope Laboratories (Andover, MA, USA); and PFOS ^13^C_4_ was obtained from Wellington Laboratories (Guelph, ON, Canada) as the internal standard (IS), and MTBE, BA were obtained from Acros (NJ, USA); ammonium acetate was procured from Alfa Aesar (Ward Hill, MA, USA). HPLC-grade methanol was purchased from Honeywell Burdick & Jackson (Morristown, NJ, USA). Deionized water from a Millipore water purification system (ELGA LabWater, High Wycombe, UK) was used to prepare all aqueous solutions. All reagents were of analytical grade.

S1.2 Serum sample preparation

The serum samples were prepared according to the published method [S1], and the preparation procedure is shown in Figure S1. In brief, serum samples were thawed at room temperature, and 250 uL of serum were mixed with 250 uL of distilled water in a 15 mL PP tube (containing 5 ng ISs). Before extraction, the spiked samples were allowed to equilibrate overnight at 4 °C. Then, 1 mL of TBA (0.5 M) and 2 mL of Na_2_CO_3_ (0.25 M, pH 10) were added into. After mixing, 5 mL of MTBE was added, and the mixture was shaken at 250 r/min for 15 min. The organic and the aqueous layers were separated by centrifugation at 1000 g for 15 min. Then, 4 mL of MTBE supernatant was taken out and transferred to another 15 mL PP tube. The procedure was repeated twice as mentioned above, except that 5 mL of MTBE was collected each time. All three extracts were combined, and evaporated to dryness under a gentle stream of nitrogen at 45 °C. Finally, the dried residue was resuspended in 250 uL of methanol/water (50:50, v/v) before analysis. Calibration standards and QC samples were analyzed concurrently with unknown samples using the similar sample preparation procedure.


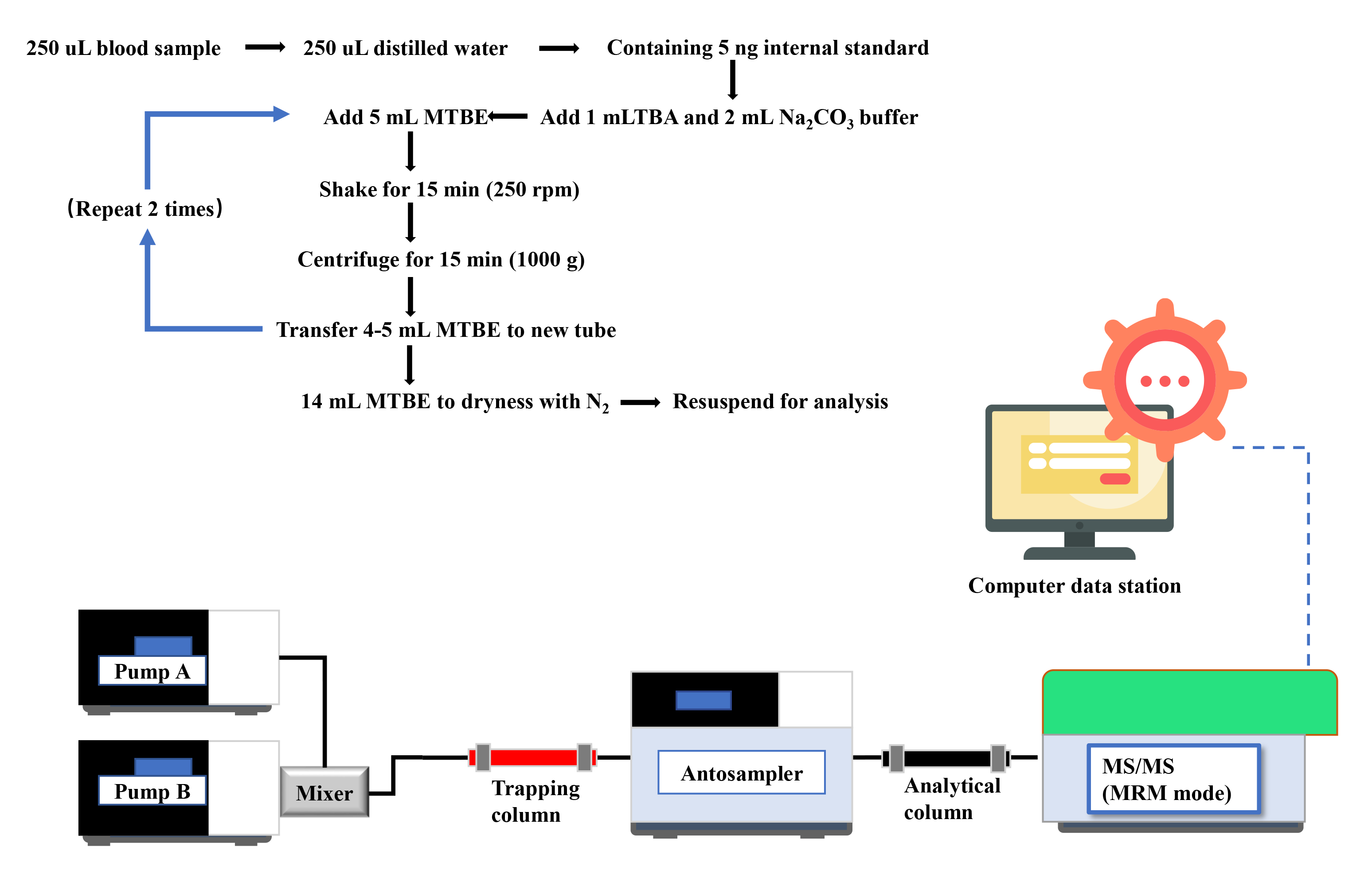


**Figure S1. Sample preparation and detection procedure of serum PFCs.**

**S1.3 LC-MS/MS analysis**

The PFCs in serum samples were measured by LC-MS/MS method proposed by the published work [S2]. The target compounds were separated using LC-MS/MS8050 (Shimadzu, Kyoto, Japan), and Accucore C_18_ column (100 mm × 4.6 mm internal diameter, 2.6 μm particle size, Thermoscientific). The gradient elution procedure of the mobile phase is shown in Table S1. After washing with a mobile phase composition of A/B (2:98) for 3.5 min, the column was equilibrated with the initial mobile phase for 3.5 min before the next injection. The total run time for each injection was 12.00 min and the injection volume were 5 uL. The mass spectrometry conditions are as follows: desolvation line temperature, 250 °C; interface temperature, 300 °C; heat block temperature, 400 °C; heating gas, 10.0 L/min; nebulizing gas, 3.0 L/min; and drying gas, 10.0 L/min. Mass spectrometry analysis was carried out in MRM mode under negative ion conditions. Optimal settings for chromatographic compound dependent retention time and MS/MS parameters for target compounds is shown in Table S2.

**Table S1. LC-MS/MS analytical mobility elution procedure**

| **Time (min)** | **Flow (mL/min)** | **A (%)** | **B (%)** |
| --- | --- | --- | --- |
| 0.01 | 0.40 | 80 | 20 |
| 5.00 | 0.40 | 0 | 100 |
| 9.00 | 0.40 | 0 | 100 |
| 9.50 | 0.40 | 80 | 20 |
| 12.00 | 0.40 | 80 | 20 |

A: 2 mM ammonium acetate in water; B: methanol

**Table S2. Retention time, transitions and MS/MS conditions of the analytes**

| **Analyte** | **Molecular mass (m/z)** | **Precursor ion (m/z)** | **Product ion (m/z)** | **Retention time (min)** | **Collision Energy** | **Dwell time (ms)** | |
| --- | --- | --- | --- | --- | --- | --- | --- |
| *PFOA | 414.07 | 413.05 | 369.00 | 6.83 | 11.0 | | 20 |
| PFOA | 414.07 | 413.05 | 169.05 | 6.72 | 19.0 | | 20 |
| PFHA | 314.05 | 313.05 | 269.05 | 6.30 | 10.0 | | 20 |
| *PFNA | 464.08 | 463.05 | 419.00 | 7.05 | 12.0 | | 20 |
| PFNA | 464.08 | 463.05 | 218.90 | 6.93 | 19.0 | | 20 |
| *PFDA | 514.08 | 513.05 | 468.95 | 7.22 | 13.0 | | 20 |
| PFDA | 514.08 | 513.05 | 218.95 | 7.21 | 18.0 | | 20 |
| *PFUnDA | 564.09 | 563.10 | 519.00 | 7.38 | 13.0 | | 20 |
| PFUnDA | 564.09 | 563.10 | 269.10 | 7.37 | 20.0 | | 20 |
| *PFHpA | 364.06 | 363.05 | 319.10 | 6.60 | 11.0 | | 20 |
| PFHpA | 364.06 | 363.05 | 119.20 | 6.49 | 22.0 | | 20 |
| *PFDoDA | 614.10 | 613.10 | 568.95 | 7.50 | 14.0 | | 20 |
| PFDoDA | 614.10 | 613.10 | 169.20 | 7.39 | 29.0 | | 20 |
| *PFOS | 500.00 | 499.00 | 80.00 | 7.02 | 49.0 | | 20 |
| PFOS | 500.00 | 499.00 | 99.05 | 6.91 | 41.0 | | 20 |
| PFBS | 300.00 | 299.00 | 80.00 | 5.90 | 35.0 | | 20 |
| *TFHSA | 400.00 | 399.00 | 79.95 | 6.58 | 46.0 | | 20 |
| TFHSA | 400.00 | 399.00 | 99.00 | 6.49 | 35.0 | | 20 |
| PFBA | 214.04 | 213.05 | 169.00 | 5.16 | 12.0 | | 20 |

“*” was used for quantitative and the second one was used for qualitative.

**S2. WGCNA procedure**

Firstly, we calculated the adjacency (Adj) between metabolites and constructed a topological overlap matrix (TOM). We then produced a hierarchical clustering tree with the dissimilarity of the TOM and we selected the modules by using the dynamic tree cut. Finally, we merged similar modules by calculating the module eigenmetabolites (ME), clustering them and assigning a distance threshold (cut of 0.2). The parameters used were soft-threshold powers and minimum module size of 3 metabolites. The Adj is calculated as follows:

Adj: ${a_{\mathrm{ij}} = \left| \mathrm{cor}\left( xi, xj \right) \right|}^{\beta}$

Specifically, $a_{ij}$ represents the adjacency of an unsigned network. The parameter β was soft-threshold powers determined by the criterion that the resulting Adj matrix approximately fits a scale-free topological feature according to a model-fitting index [S3]. The optimal β values for CPP and PT in this study were 9 and 7, respectively (Figure S2A**)**.

Once the unsigned network was constructed, the densely interconnected metabolites clusters modules were detected based on the topological overlap measure (TOM) matrix and unsupervised cluster (the default method is hierarchical clustering). The TOM matrix was converted from an aij according to the formula:

$$\mathrm{TOM}_{\mathrm{ij}}=\frac{\sum_{u\neq\mathrm{ij}} {a_{\mathrm{iu}}a}_{\mathrm{uj}}+a_{\mathrm{ij}}}{\min\left( k_{i}+k_{j} \right)+1-a_{\mathrm{ij}}}$$

Where k_i_ = ∑_u≠i_ a_ui_ is the node connectivity (known as degree). For each metabolite, the connectivity is defined as the sum of connection strengths with the other network metabolites. In co-expression networks, the connectivity measures how correlated a metabolite is with all other network metabolites.

Identification of biologically significant modules and metabolite is one major goal of co-expression analysis [S4]. The WGCNA approach defines a *GS* that assigns a nonnegative number to each metabolite; the higher *GS*_i_ the more biologically significant is metabolite *i*. Sample trait *T* (represent: clinical phenotype and PFCs) can be used to define a trait-based *GS* as the absolute correlation between the traits and the expression profiles. Then, the important modules were identified according to the correlation value and p value of the correlation test between the modules and clinical traits (|cor| > 0.30 and *p* < 0.05).

$${GS}_{i} = \left| \mathrm{cor}\left( xi,T \right) \right|$$




**Figure S2. WGCNA related visualization diagram. (**A): Analysis of metabolomics network topology for various soft-thresholding powers in CPP (a1) and PT (a2), the scale-free fit index (y-axis) as a function of the soft thresholding power (x-axis). (B): Clustering dendrograms of metabolites in CPP (b1) and PT (b2), with dissimilarity based on topological overlap, together with assigned module colors. There is one metabolite dendrogram per block. (C): Eigenmetabolites adjacency heatmap in CPP (c1) and PT (c2).


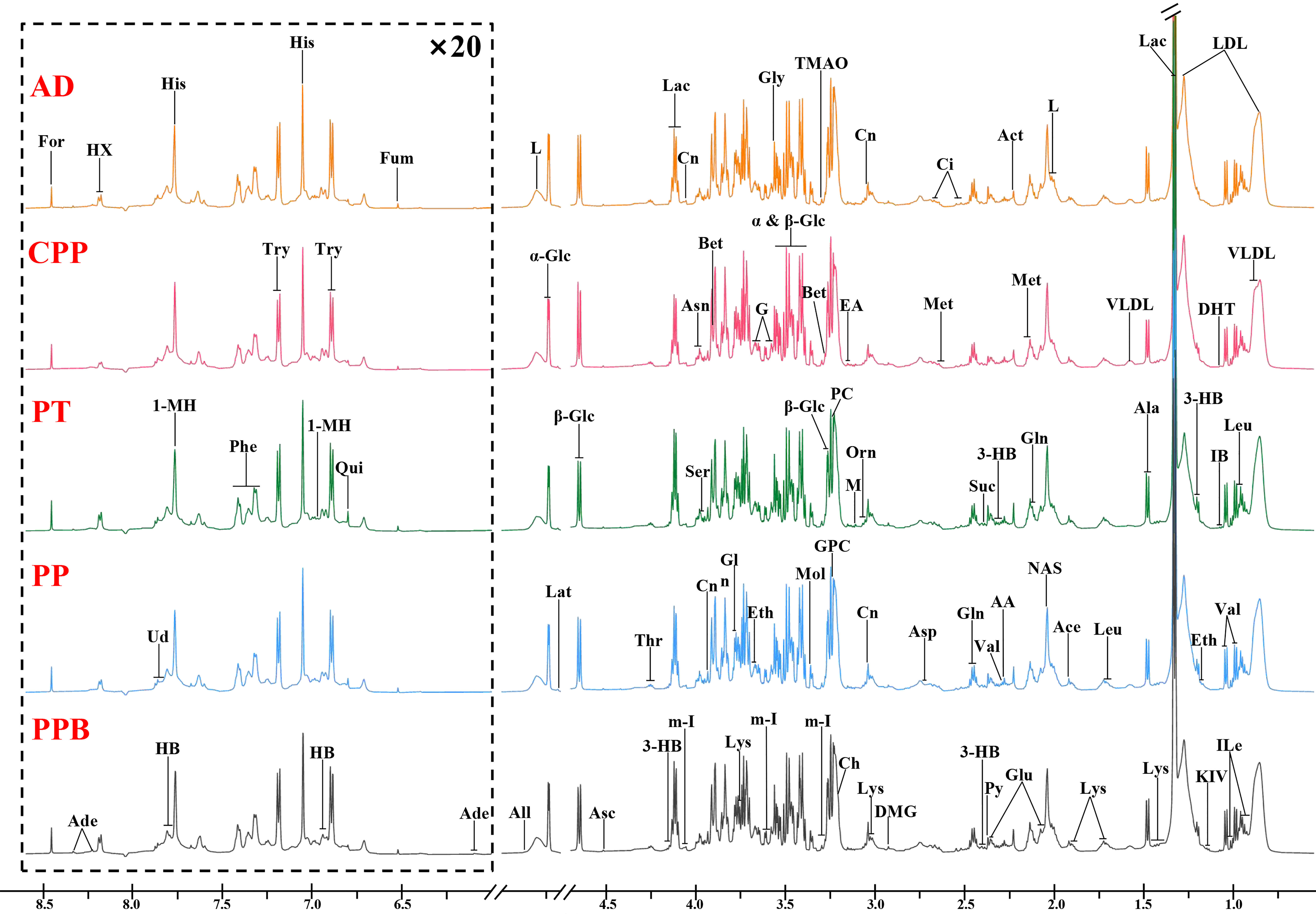


**Figure S3. Mean ^1^H-NMR spectra of serum from prepubertal, PP, PT, CPP and adolescent girls**. The spectral regions of δ6.00-8.60 (in the dashed box) were vertically expanded 20 times compared with those of δ0.50-6.00 for the purpose of clarity, and the keys of the abbreviations on the spectra and the spectral information were represented in Table S3. PPB: Prepubertal; AD: Adolescent

**Table S3. The identified metabolites from ^1^H-NMR spectra of the girls’ serum samples**

| **Metabolites** | **Abbr** | **Chemical shift (multiplicity)** | **KEGG_ID** | **HMDB_ID** |
| --- | --- | --- | --- | --- |
| 1-Methylhistidine | 1-MH | 6.99(s1), 7.68(s)2 | C01152 | HMDB0000001 |
| 3-Hydroxybutyrate | 3-HB | 1.20(d), 4.16(m), 2.31(dd), 2.41(dd) | C01089 | HMDB0000011 |
| Acetate | Ace | 1.92(s) | C00033 | HMDB0000042 |
| Acetoacetate | AA | 2.28(s), 3.43(s) | C00164 | HMDB0000060 |
| Acetone | Act | 2.23(s) | C00207 | HMDB0001659 |
| Adenosine | Ade | 6.10(d), 8.23(s), 8.34(s) | C00212 | HMDB0000050 |
| Alanine | Ala | 1.48(d) | C00041 | HMDB0000161 |
| Allantoin | All | 5.41(m) | C01551 | HMDB0000462 |
| Ascorbate | Asc | 4.52(d) | C01041 | HMDB0000044 |
| Asparagine | Asn | 2.85(dd), 2.94(dd), 3.98(m) | C01905 | HMDB0033780 |
| Aspartate | Asp | 2.71(dd), 2.81(dd) | C00049 | HMDB0000191 |
| Betaine | Bet | 3.27(s), 3.90(s) | C00719 | HMDB0000043 |
| Choline | Ch | 3.20(s) | C00114 | HMDB0000097 |
| Citrate | Ci | 2.53(d), 2.67(d) | C00158 | HMDB0000094 |
| Creatine | Cr | 3.04(s), 3.93(s) | C00300 | HMDB0000064 |
| Creatinine | Cn | 3.05(s) , 4.05(s) | C00791 | HMDB0000562 |
| Dihydrothymine | DHT | 1.09(d) | C00906 | HMDB0000079 |
| Dihydroxyacetone | DHA | 4.42(s) | C00184 | HMDB0001882 |
| Ethanol | Eth | 1.18(t), 3.66(q) | C00469 | HMDB0000108 |
| Ethanolamine | EA | 3.15(t) | C00189 | HMDB0000149 |
| Formate | For | 8.45(s) | C00058 | HMDB0000142 |
| Fumarate | Fum | 6.52(s) | C00122 | HMDB0000134 |
| Glutamate | Glu | 2.08(m), 2.35(m) | C00302 | HMDB0060475 |
| Glutamine | Gln | 2.13(m), 2.45(m), 3.78(t) | C00064 | HMDB0000641 |
| Glycerol | G | 3.57(m), 3.66(m) | C00116 | HMDB0000131 |
| Glycerophosphoryl-choline | GPC | 3.23(s), 3.35(s), 3.68(m), 4.33(m) | C00670 | HMDB0000086 |
| Glycine | Gly | 3.56(s) | C00037 | HMDB0000123 |
| Histidine | His | 3.13(m), 7.06(s), 7.77(s) | C00135 | HMDB0000177 |
| Hypoxanthine | HX | 8.18(s), 8.20(s) | C00262 | HMDB0000157 |
| Isobutyrate | IB | 1.07(d) | C02632 | HMDB0001873 |
| Isoleucine | Ile | 0.94(t), 1.01(d) | C00407 | HMDB0000172 |
| Lactate | Lac | 1.33(d), 4.11(q) | C00186 | HMDB0000190 |
| Lactose | Lat | 5.19(d) | C00243 | HMDB0000186 |
| Leucine | Leu | 0.96(t), 1.70(m) | C00123 | HMDB0000687 |
| Lipid | L | 2.02(br), 2.24(br), 2.75(br), 5.31(br) | C13908 | HMDB0013244 |
| Low density lipoprotein | LDL | 0.85(br), 1.28(br) | —— | —— |
| Lysine | Lys | 1.43(m), 1.73(m), 1.89(m), 3.03(t), 3.76(t) | C00047 | HMDB0003405 |
| Malonate | M | 3.11(s) | C04025 | HMDB0000691 |
| Methanol | Mol | 3.36(s) | C00132 | HMDB0001875 |
| Methionine | Met | 2.14(s), 2.16(m), 2.64(t) | C00073 | HMDB0000696 |
| *myo*-Inositol | m-I | 3.30(t), 3.61(m), 4.07(t) | C00137 | HMDB0000211 |
| N, N-Dimethylglycine | DMG | 2.93(s) | C01026 | HMDB0000092 |
| N-Acetyl-glycoprotein signals | NAS | 2.04(s) | —— | —— |
| Ornithine | Orn | 3.06(t) | C00077 | HMDB0000214 |
| para-Hydroxybenzoate | HB | 6.94(d), 7.81(d) | —— | —— |
| Phenylalanine | Phe | 7.33(d), 7.36(m), 7.42(m) | C00079 | HMDB0000159 |
| Phosphocholine | PC | 3.22(s) | C00157 | HMDB0007886 |
| Pyruvate | Py | 2.37(s) | C00022 | HMDB0000243 |
| Quinone | Qui | 6.80(s) | C00472 | HMDB0003364 |
| Serine | Ser | 3.96(m) | C00065 | HMDB0000187 |
| Succinate | Suc | 2.41(s) | C00042 | HMDB0000254 |
| Threonine | Thr | 4.25(m) | C00188 | HMDB0000167 |
| Trimethylamine N-oxide | TMAO | 3.27(s) | C01104 | HMDB0000925 |
| Tyrosine | Tyr | 6.89(d), 7.19(d) | C00082 | HMDB0000158 |
| Uridine | Ud | 7.87(dd) | C00299 | HMDB0000296 |
| Valine | Val | 0.99(d), 1.04(d), 2.27(m) | C00183 | HMDB0000883 |
| Very low-density lipoprotein | VLDL | 0.89(br), 1.31(br), 1.58(br) | —— | —— |
| α-Glucose | α-Glc | 3.42(t), 3.54(dd), 3.72(t), 3.84(m), 5.24(d) | C00221 | HMDB0000122 |
| α-Ketoisovalerate | KIV | 1.14(d) | C00141 | HMDB0000019 |
| β-Glucose | β-Glc | 3.25(dd), 3.41(t), 3.46(dd), 3.49(t), 3.90(dd), 3.73(t), 4.65(d), | C00221 | HMDB0000122 |

^1^ Multiplicity: s, singlet; d, doublet; t, triplet; q, quartet; dd, doublet of doublets; m, multiplet.

^2^ Underlined chemical shift represents the characteristic peak of the metabolite for quantitative analysis.

**Table S4. Summary of the quality parameters of the multivariate statistical analysis**

| **Model** | **Group** | **R^2^X** | **R^2^Y** | **Q^2^** | **Permutations (200)** | |
| --- | --- | --- | --- | --- | --- | --- |
|  |  |  |  |  | **R^2^ intercepts** | **Q^2^ intercepts** |
| OPLS-DA | PP *vs*. prepubertal girls | 0.673 | 0.446 | 0.242 | 0.168 | －0.239 |
|  | PP *vs*. adolescent girls | 0.693 | 0.469 | 0.316 | 0.186 | －0.246 |
|  | CPP *vs*. prepubertal girls | 0.368 | 0.802 | 0.412 | 0.702 | －0.380 |
|  | CPP *vs.* adolescent girls | 0.380 | 0.815 | 0.525 | 0.736 | －0.419 |
|  | PT *vs.* prepubertal girls | 0.341 | 0.715 | 0.289 | 0.658 | －0.439 |
|  | PT *vs.* adolescent girls | 0.352 | 0.807 | 0.520 | 0.678 | －0.498 |
| PLS-DA | CPP *vs*. prepubertal girls | 0.675 | 0.998 | 0.996 | —— | —— |
|  | CPP *vs*. prepubertal＋adolescent girls | 0.61 | 0.997 | 0.995 | —— | —— |
|  | PT *vs*. prepubertal girls | 0.709 | 0.99 | 0.977 | —— | —— |
|  | PT *vs*. prepubertal＋adolescent girls | 0.677 | 0.996 | 0.992 | —— | —— |

**Table S5. The potential biomarkers in serum of PP**

| **Potential biomarker** | **Raw *p* value** | **VIP** | **FC** | **Age-adjusted** | |
| --- | --- | --- | --- | --- | --- |
|  |  |  |  | **OR (95% Cl)** | **p-adj** |
| **Compared with the prepubertal girls** | | | | | |
| Creatine | 1.69E-02 | 2.180 | 0.939 | 0.175 (0.160 to 0.189) | 7.76E-01 |
| Dihydroxyacetone | 6.43E-03 | 2.644 | 1.156 | 0.017 (0.009 to 0.240) | 7.64E-03 |
| Ethanolamine | 7.68E-03 | 1.711 | 1.071 | 0.066 (0.059 to 0.073) | 1.17E-02 |
| Glutamine | 3.30E-02 | 4.932 | 1.056 | 1.131 (1.006 to 1.257) | 9.10E-02 |
| Lactose | 3.28E-03 | 1.533 | 1.164 | 0.018 (0.009 to 0.026) | 4.08E-02 |
| Methanol | 1.50E-02 | 3.253 | 1.118 | 0.062 (0.048 to 0.075) | 3.36E-02 |
| *para*-Hydroxybenzoate | 3.60E-02 | 1.258 | 1.059 | 0.022 (0.026 to 0.025) | 3.42E-02 |
| Quinone | 2.42E-02 | 1.075 | 1.297 | 0.002 (0.000 to 0.004) | 1.02E-01 |
| Trimethylamine *N*-oxide | 8.63E-03 | 2.785 | 1.084 | 0.011 (0.009 to 0.012) | 2.04E-02 |
| **Compared with the adolescent girls** | | | | | |
| 3-Hydroxybutyrate | 9.49E-07 | 6.377 | 1.279 | 0.096 (0.036 to 0.185) | 4.33E-02 |
| Acetate | 6.28E-03 | 2.037 | 1.084 | 0.081 (0.064 to 0.098) | 3.30E-04 |
| Acetoacetate | 1.71E-03 | 2.552 | 1.121 | 0.068 (0.043 to 0.094) | 2.34E-02 |
| Acetone | 1.21E-02 | 5.275 | 1.189 | 0.019 (-0.010 to 0.048) | 9.23E-02 |
| Alanine | 4.45E-02 | 5.492 | 0.924 | 0.456 (0.399 to 0.573) | 3.47E-02 |
| Choline | 1.36E-02 | 3.913 | 1.084 | 0.087 (0.070 to 0.105) | 8.62E-01 |
| Creatine | 2.99E-03 | 1.190 | 1.078 | 0.182 (0.148 to 0.215) | 6.49E-01 |
| Ethanol | 1.80E-04 | 6.178 | 1.225 | 0.111 (-0.015 to 0.236) | 1.32E-01 |
| Isoleucine | 3.19E-03 | 2.564 | 0.928 | 0.136 (0.112 to 0.160) | 3.90E-04 |
| Lipid | 2.43E-03 | 2.649 | 0.937 | 1.601 (1.298 to 1.905) | 2.40E-04 |
| Serine | 2.36E-03 | 2.629 | 1.089 | 0.170 (0.137 to 0.204) | 4.60E-04 |
| Succinate | 7.60E-03 | 1.870 | 1.099 | 0.019 (0.013 to 0.025) | 2.16E-02 |
| Tyrosine | 2.23E-02 | 2.040 | 0.909 | 0.078 (0.062 to 0.094) | 1.80E-04 |
| α-Ketoisovalerate | 1.19E-02 | 1.550 | 0.851 | 0.023 (0.015 to 0.030) | 4.84E-02 |

FC: fold change of metabolite, FC = C disease/C control, where FC > 1 means elevated content and FC < 1 indicates decreased content of metabolite.

**Table S6. Linear range, correlation coefficient, LOD and recovery of PFCs (n = 5).**

| **Abbreviation** | **Linearity range (μg/L)** | **Correlation coefficent (r^2^)** | **LOD (μg/L)** | **Recovery (%)** |
| --- | --- | --- | --- | --- |
| PFOA | 0-100 | 0.99 | 0.12 | 86.0 |
| PFOS | 0-100 | 0.99 | 0.09 | 107 |
| PFBA | 0-100 | 0.99 | 0.09 | 78.0 |
| PFUnDA | 0-100 | 0.99 | 0.24 | 124 |
| PFDoDA | 0-100 | 0.99 | 0.09 | 73.0 |
| PFBS | 0-100 | 0.99 | 0.10 | 91.0 |
| PFDA | 0-100 | 0.99 | 0.06 | 81.0 |
| PFHpA | 0-100 | 0.99 | 0.09 | 113 |
| PFHA | 0-100 | 0.99 | 0.02 | 70.0 |
| TFHSA | 0-100 | 0.99 | 0.14 | 86.0 |
| PFNA | 0-100 | 0.99 | 0.08 | 83.0 |

LOD: Limit of detection

**Table S7. Concentration and statistical analysis of serum PFCs in the CPP and PT groups**

| **PFCs** | **Concentration (ng/mL)** | | **Mann-Whitney (*p*-value**) |
| --- | --- | --- | --- |
|  | **CPP (n = 30)** | **PT (n = 40)** |  |
| PFOA | 5.177 ± 1.98 | 5.921 ± 2.48 | 2.03E-01 |
| PFOS | 10.20 ± 10.1 | 9.185 ± 6.69 | 6.96E-01 |
| PFBA | 0.186 ± 0.30 | 0.322 ± 0.43 | 1.62E-01 |
| PFUnDA | 0.507 ± 0.38 | 0.539 ± 0.27 | 3.01E-01 |
| PFDoDA | 0.155 ± 0.17 | 0.163 ± 0.12 | 6.90E-01 |
| PFBS | 0.051 ± 0.1 | 0.080 ± 0.1 | 8.00E-02 |
| PFDA | 0.868 ± 0.55 | 0.920 ± 0.49 | 5.37E-01 |
| PFHpA | 0.355 ± 1.3 | 0.261 ± 0.29 | 2.30E-02* |
| PFHA | 2.635 ± 9.80 | 1.110 ± 0.823 | 4.70E-02* |
| TFHSA | 0.702 ± 0.43 | 0.684 ± 0.64 | 4.05E-01 |
| PFNA | 1.289 ± 0.550 | 1.521 ± 0.675 | 1.77E-01 |

“*” represents a significantly statistical difference between the CPP and PT.


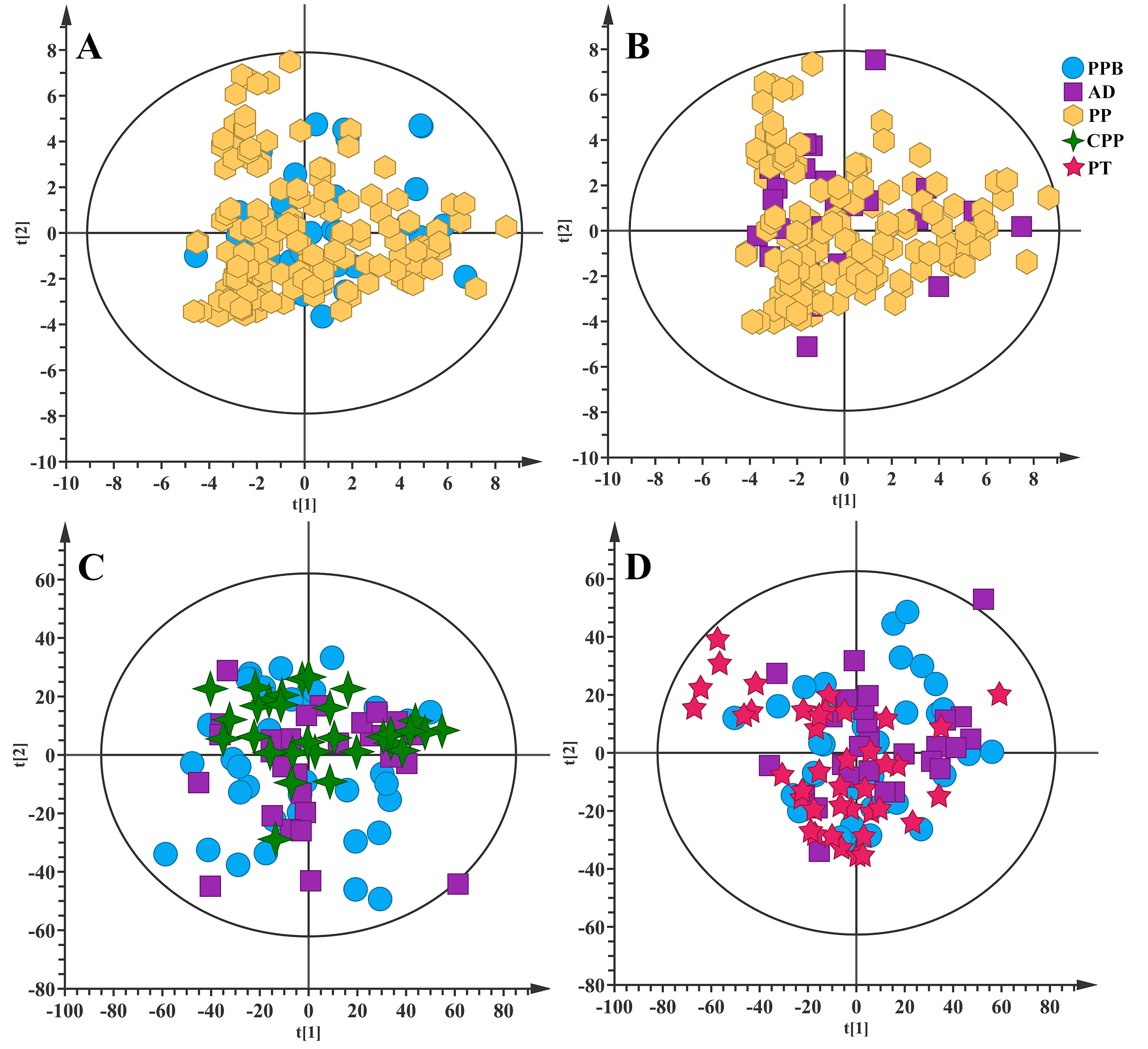


**Figure S4. PCA score plots of serum samples.** (A) the PP and prepubertal girls; (B) the PP and adolescent girls; (C) the CPP, prepubertal and adolescent girls; (D) the PT, prepubertal and adolescent girls. The sample numbers of prepubertal, adolescent, PP, CPP and PT girls were 36, 28, 146, 30 and 40, respectively. PPB: Prepubertal; AD: Adolescent





**Figure S5. Permutation test analysis to test the over-fitting of OPLS-DA model, and the evaluation indexes of intercepts R^2^ and Q^2^ are listed in the Table S4.** PP: Precocious puberty; CPP: Central precocious puberty; PT: Premature thelarche; PPB: Prepubertal; AD: Adolescent


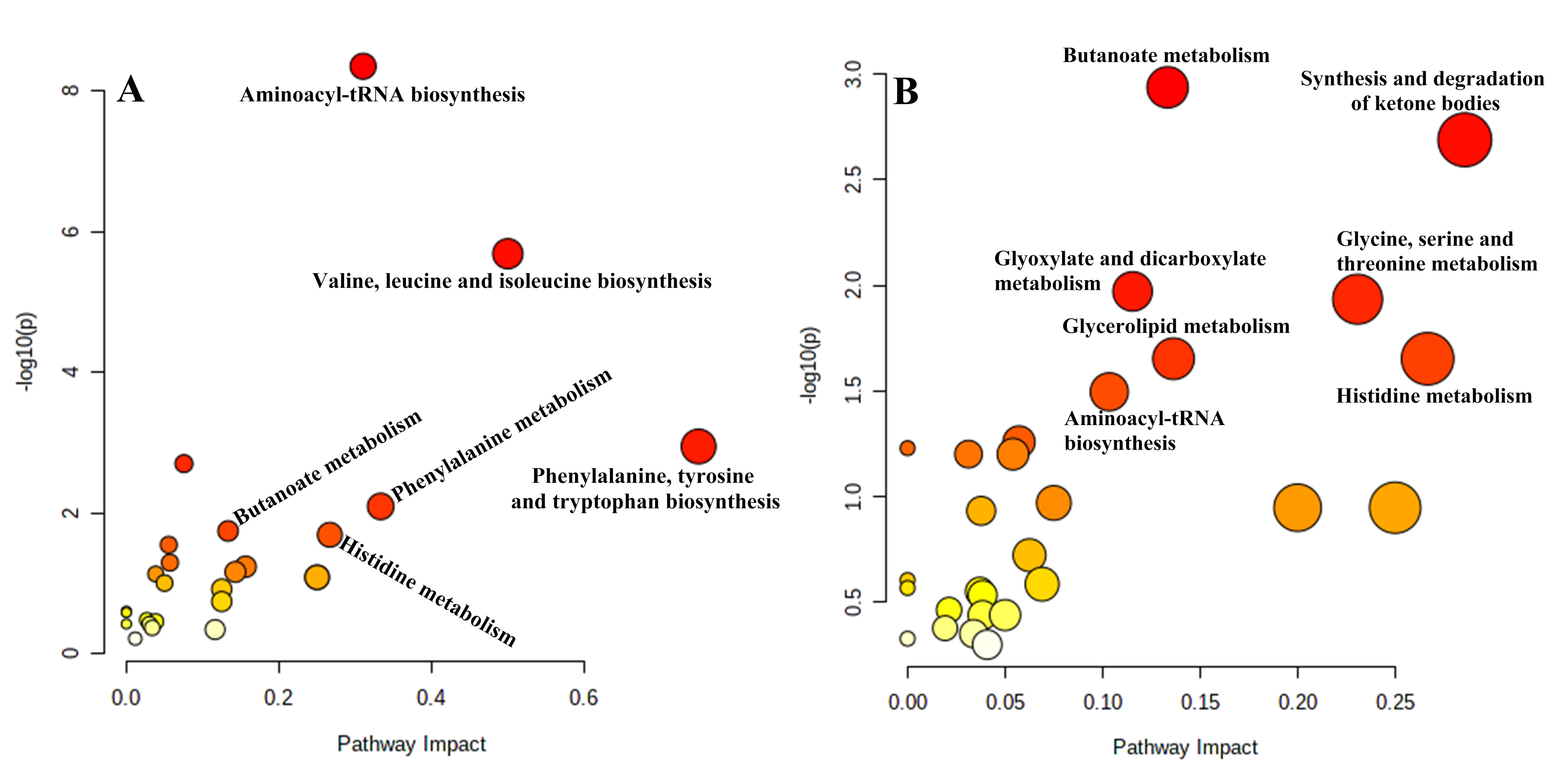


**Figure S6. The pathways enrichment analysis of the CPP (A) and PT (B) base on the potential biomarkers via MetaboAnalyst 5.0.** Each point represents one metabolic pathway; the size of dot and shades of color are in positive correlation with the impact of the metabolic pathways.

**References**

S1. Liu L, She J, Zhang X, Zhang J, Tian M, Huang Q, Shah Eqani SA, Shen H: Online background cleanup followed by high-performance liquid chromatography with tandem mass spectrometry for the analysis of perfluorinated compounds in human blood. *J Sep Sci* 2015, 38(2):247-253.

S2. Yusa V, Ye X, Calafat AM: Methods for the determination of biomarkers of exposure to emerging pollutants in human specimens. *Trends Analyt Chem* 2012, 38:129-142.

S3. Zhang B, Horvath S: A general framework for weighted gene co-expression network analysis. *Stat Appl Genet Mol Biol* 2005, 4:Article17.

S4. Pei G, Chen L, Zhang W: WGCNA Application to Proteomic and Metabolomic Data Analysis. *Methods Enzymol* 2017, 585:135-158.
